# INPP5D regulates inflammasome activation in human microglia

**DOI:** 10.1038/s41467-023-42819-w

**Published:** 2023-11-29

**Authors:** Vicky Chou, Richard V. Pearse, Aimee J. Aylward, Nancy Ashour, Mariko Taga, Gizem Terzioglu, Masashi Fujita, Seeley B. Fancher, Alina Sigalov, Courtney R. Benoit, Hyo Lee, Matti Lam, Nicholas T. Seyfried, David A. Bennett, Philip L. De Jager, Vilas Menon, Tracy L. Young-Pearse

**Affiliations:** 1 Ann Romney Center for Neurologic Diseases, Department of Neurology, Brigham and Women’s Hospital and Harvard Medical School, Boston, MA USA; 2 Center for Translational and Computational Neuroimmunology, Department of Neurology, and the Taub Institute for the Study of Alzheimer’s Disease and the Aging Brain, Columbia University Irving Medical Center, New York, NY USA; 3 Department of Biochemistry, Emory School of Medicine, Atlanta, GA USA; 4 Department of Neurology, Emory School of Medicine, Atlanta, GA USA; 5 Rush Alzheimer’s Disease Center, Rush University Medical Center, Chicago, IL USA; 6 Harvard Stem Cell Institute, Harvard University, Cambridge, MA USA

**Keywords:** Alzheimer's disease, Neuroimmunology, Cellular neuroscience, Microglia

## Abstract

Microglia and neuroinflammation play an important role in the development and progression of Alzheimer’s disease (AD). Inositol polyphosphate-5-phosphatase D (*INPP5D/SHIP1*) is a myeloid-expressed gene genetically-associated with AD. Through unbiased analyses of RNA and protein profiles in INPP5D-disrupted iPSC-derived human microglia, we find that reduction in INPP5D activity is associated with molecular profiles consistent with disrupted autophagy and inflammasome activation. These findings are validated through targeted pharmacological experiments which demonstrate that reduced INPP5D activity induces the formation of the NLRP3 inflammasome, cleavage of CASP1, and secretion of IL-1β and IL-18. Further, in-depth analyses of human brain tissue across hundreds of individuals using a multi-analytic approach provides evidence that a reduction in function of INPP5D in microglia results in inflammasome activation in AD. These findings provide insights into the molecular mechanisms underlying microglia-mediated processes in AD and highlight the inflammasome as a potential therapeutic target for modulating INPP5D-mediated vulnerability to AD.

## Abstract (web summary)

INPP5D/SHIP1 is a microglial-expressed gene that has been associated with Alzheimer’s disease through genetic studies. This study reveals that reduction in INPP5D activity induces activation of the NLRP3-inflammasome in human microglia.

## Introduction

There is an increasing focus on understanding the role of microglia in neurodegenerative disorders including late-onset Alzheimer’s disease (LOAD), a disease defined by the accumulation of amyloid beta rich plaques and neurofibrillary tangles. Microglia play a variety of key roles during disease progression through synaptic engulfment^1^, cytokine release, and phagocytosis of amyloid beta^2^. Further, recent genome-wide association studies (GWAS) have implicated innate immune processes in LOAD, supporting the importance of understanding the neuroimmunological processes underlying the risk and progression of AD. However, in many cases the molecular mechanisms underlying the consequences of perturbation of AD-associated genes in microglia are poorly understood.

The inflammasome is a multimeric complex involved in innate immune signaling that is induced in response to inflammatory insults^3^. Inflammasome activation involves conformational changes in NLR family members that allow for association and oligomerization with the adapter protein ASC (PYCARD) and inactive pro-caspase-1 (CASP1). Upon inflammasome formation, pro-CASP1 is cleaved to generate active CASP1 which in turn cleaves pro-IL-1ß and pro-IL-18 resulting in extracellular release of these cytokines^3^. Inflammasome activity is associated with several inflammatory disorders and more recently with AD^4–6^. In mouse models of familial AD, inflammasome activation in microglia has been shown to contribute to amyloid beta and tau pathology^4,5^. The regulation of inflammasome activation in human microglia is poorly understood, thus the identification of other members of this signaling cascade would prove valuable towards targeting inflammasome activity in disease.

Interrogating the connections between genetic risk factors for AD and inflammatory cascades will be important for identifying new therapeutic strategies for AD. One such genetic risk factor highly expressed in myeloid cells is INPP5D. INPP5D was first characterized through loss-of-function mutations that cause leukemia and other related myeloproliferative diseases^7^. Subsequent studies revealed that loss of INPP5D expression in peripheral macrophages resulted in dysregulation of immune reactivity^8^. INPP5D encodes a 145 kDa membrane-associated phosphatase that acts as a negative regulator of PI3K/AKT signaling by hydrolyzing the 5′-phosphate of the secondary messenger, phosphatidylinositol (3,4,5)P_3_ to generate phosphatidylinositol (3,4)P_2_^9^. Elevated INPP5D activity results in reduced levels of phosphorylated AKT, which in turn affects cell metabolism and survival signaling^10^. INPP5D knockout mouse models experience an over-proliferation of peripheral myeloid cells and early postnatal lethality^8^. In addition, INPP5D has been reported to function as a scaffolding protein, binding to DAP12 (DNAX-activating protein of 12 kDa) to prevent PI3K association with TREM2 (triggering receptor expressed on myeloid cells 2)^11^. INPP5D also influences phagocytic activity of macrophages through binding to FcγR and ROS production through interactions with Dectin-1/CLEC7A^12,13^. Studies of decreased microglia INPP5D expression in AD mouse models have found seemingly conflicting results. In one study decreased INPP5D expression increased plaque clearance^14^ while in another shows that INPP5D deficiency exacerbated plaque burden^15^, suggesting that INPP5D function is complex and likely context-dependent.

Single nucleotide polymorphisms (SNPs) at the *INPP5D* locus have been associated with LOAD through GWAS^16–19^. The lead SNPs, rs10933431, rs35349669, and rs7597763, are each located within introns of *INPP5D*. A recent study revealed that these SNPs are associated with differential promoter utilization and splicing. This results in forms of INPP5D that lack the phosphatase domain^20^. Studies to date have not provided clarity as to whether AD risk SNPs are associated with higher or lower INPP5D activity.

Here, we show that INPP5D expression is largely restricted to microglial cells in the human adult brain and confirm previous findings that RNA levels of INPP5D are elevated in AD brain^21^. Quantitative immunocytochemistry revealed that a pool of INPP5D protein is elevated in plaque-associated microglia, however, quantitative western blotting reveals that protein levels of full length, aqueous INPP5D are *reduced* in AD brain. To study the functional consequences of reduced INPP5D, we use both pharmacological inhibition and CRISPR-Cas9 genome engineering to reduce INPP5D activity in human iPSC-derived microglia. Through unbiased RNA and proteomic profiling and a series of pharmacological manipulations, we demonstrate that reduction of INPP5D activity in microglia induces changes in immune signaling and, more specifically, the activation of the NLRP3 inflammasome. Returning to analyses of human brain tissue, we provide evidence of linkage between INPP5D levels and inflammasome activation in the AD brain. By profiling both a biallelic loss of function and overexpression model of INPP5D, we find that microglia in the AD brain most closely resemble the loss-of-function iMG model with regard to effects on immune pathways, supporting the hypothesis that microglia in the AD brain have reduced INPP5D function. Finally, through a co-culture experimental model system, we identify functional consequences of reduced INPP5D in microglia on neuronal synaptic gene expression.

## Results

### INPP5D expression is enriched in human microglia

**Fig. 1:**
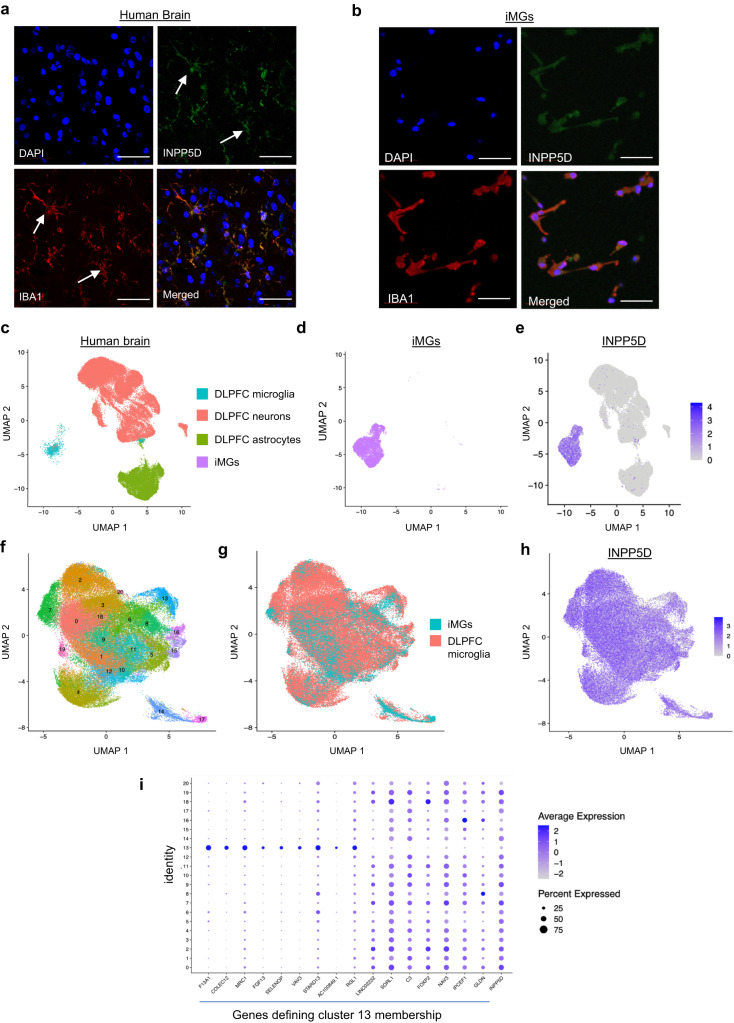
INPP5D expression in the human brain is restricted to microglia in the human brain. Human brain sections (25 μm) (**a**) and cultured iMGs (**b**) were immunostained for IBA1 and INPP5D. Nuclei are visualized with DAPI and preparations were imaged using confocal microscopy. Images representative of 16 human brain samples and over three iMG differentiations analyzed. Scale bars = 50 μm. **c–e** UMAP plots of sNucRNAseq of iMGs combined with snRNAseq of dorsolateral prefrontal cortex (dlpfc) from 12 human postmortem brain samples^29^ using Harmony^35^ to integrate across datasets. Single nucleus data from human brain samples and iMGs are depicted separately in (**c**) and (**d**), respectively. Relative INPP5D expression (after applying the SCTransformation in the Seurat package) across the harmonized iMG and human brain single nucleus samples shown in (**e**). Data are from 7121 iMG nuclei, 55,671 nuclei from the postmortem human brain (39,239 glutamatergic neurons, 14,958 astrocytes, 1474 microglia). **f–i** Data from microglia subcluster from the snRNAseq in **c** and **d** were isolated and re-clustered to examine microglial subsets. UMAP plots are shown in (**f–h**). Relative *INPP5D* expression (z-score of log-transformed, normalized data) across the subclusters is shown in (**h**). *INPP5D* was significantly lower in cluster 13 (adj. *p*-val = 8.0 × 10^−226^, Wilcoxon rank-sum test, with FDR correction). A dot plot showing a subset of the genes that define cluster 13 are shown in (**i**). See also Supplementary Fig. 1 and Supplementary Data 1 showing enrichments in all microglial clusters.

We first confirmed INPP5D expression in human microglia using immunostaining. Human post-mortem brain tissue was fixed, cryosectioned, and immunostained for INPP5D and IBA1, a microglial and macrophage-specific calcium-binding protein. INPP5D and IBA1 co-localized in the same subset of cells, suggesting that INPP5D is expressed in microglia in the human brain (Fig. 1a).

We next established a manipulable human experimental system for assaying INPP5D function in human microglia. This was accomplished by adapting an existing published protocol that generates iPSC-derived microglia-like cells (iMGs) after 40 days of differentiation (Supplementary Fig. 1a, b)^22,23^. INPP5D expression in iMGs was confirmed using immunocytochemistry with antibodies to INPP5D and IBA1, with the two signals co-localizing to >99% of the resultant cells (Fig. 1b). Using established protocols, we also generated iPSC-derived astrocytes (iAs)^24^, induced neurons (iNs)^25,26^, and iPSC-derived endothelial cells (iECs)^27,28^ and evaluated INPP5D expression in each of these cell types using western blotting. INPP5D protein expression was restricted to iMGs (Supplementary Fig. 1c).

To further confirm the utility of this model for studying INPP5D function, we performed single-nucleus RNA sequencing (snRNAseq) to compare the genetic signatures of the iMGs to snRNAseq of human post-mortem dorsolateral prefrontal cortex (DLPFC) brain tissue from 12 adult individuals from the Religious Order Study (ROS) and Rush Memory and Aging Project (MAP) (6 male and 6 female, ranging in age at death of 78–98 years, data from ref. ^29^)^30–34^. Data from astrocytes, glutamatergic neurons, and microglia from brain tissue were merged and integrated with data from the iMGs samples using Harmony (v.0.1.1)^35^. The iMG cluster overlapped in UMAP space with the human microglia and not with neurons and astrocytes, suggesting transcriptional similarity between iMGs and human brain microglia (Fig. 1c, d). Examination of *INPP5D* expression within these populations confirmed that expression is largely confined to the brain tissue microglia and iMG cluster (Fig. 1e, Supplementary Fig. 1d). We next re-clustered data from nuclei only from the microglia cluster (Fig. 1f–h). INPP5D is expressed throughout all subclusters of microglia but is significantly lower in cluster 13 (see Supplementary Data 1 for complete analyses). Genes defining membership in cluster 13 are shown (Fig. 1i, Supplementary Data 13). Cluster 13 is enriched in genes shown to be upregulated in border associated macrophages such as F13A1, MRC1, COLEC12 and CD163. Taken together, these data demonstrate that INPP5D expression is enriched in microglia in the human brain and suggest that it is expressed at varying levels in different brain myeloid cell subtypes.

### Immunocytochemistry reveals elevated levels of INPP5D puncta in AD microglia in the brain

**Fig. 2:**
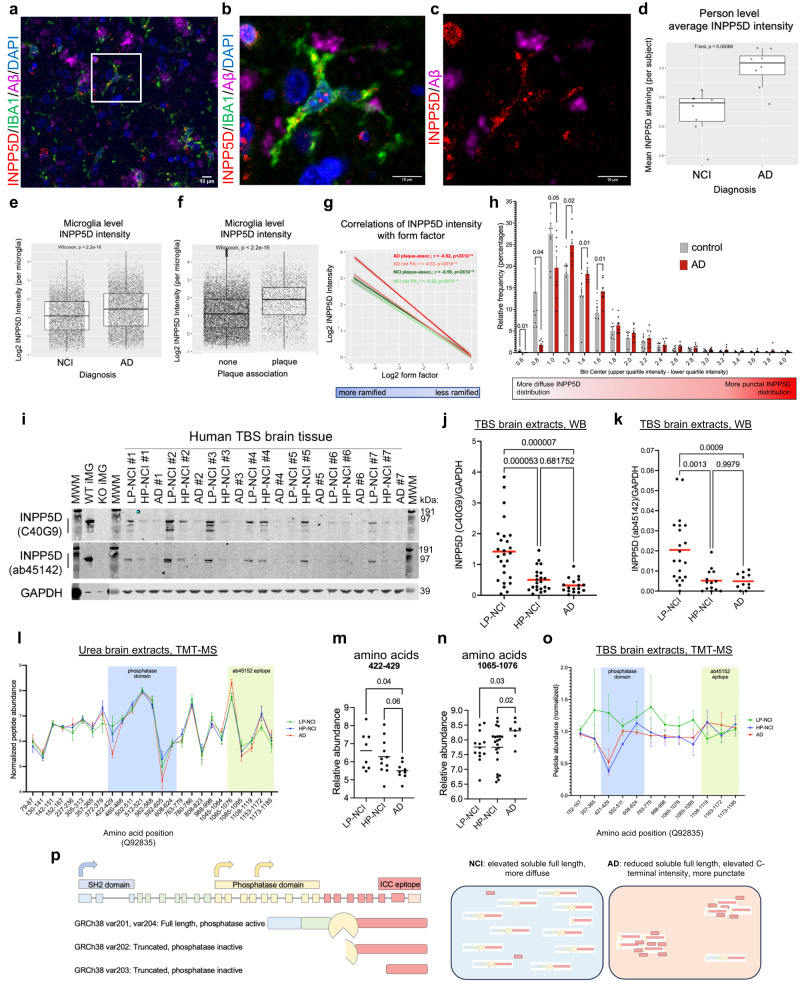
Complex post-translational regulation of INPP5D in the AD brain. **a–c** Representative images of immunostaining of human brain sections for INPP5D, IBA1, and Aβ. DNA is stained with DAPI. Scale bars = 10 μm. **b**, **c** Magnified image from white box outlined in (**a**). **d**–**h** Quantifications from brain immunostaining from 8 NCI and 8 AD individuals. **d** Quantification of intensity of INPP5D immunostaining across human subjects, two-sided *t*-test, *p* = 0.0009. **e** Quantification of INPP5D intensity across all microglia quantified between the 16 human subjects. Two-sided Wilcoxon test, *p* < 2.2e−16. **f** Quantification of INPP5D levels in microglia that were either plaque-associated (plaque) or not plaque-associated (none). Two-sided Wilcoxon test, *p* < 2.2e-16. For **d–f**, boxes mark the 1st and 3rd quartiles of data (25th and 75th percentiles), surrounding the median. Whiskers extend to furthest values up to 1.5xIQR (inter-quartile range or distance between 1st and 3rd quartiles). Data beyond the whiskers are “outlying” points for the purpose of plotting and extend to the min and max of individual datasets. **g** Correlations between form factor and INPP5D intensity across categories of microglia; regression lines for individual datasets as well as the *r*- and *p*-value statistics, represent the output of linear model calculation in R. Shaded regions represent 95% CI limits for each dataset. **h** Histogram of the relative frequency of IBA1+ microglia containing diffuse versus punctal INPP5D immunostaining intensity, determined by the difference between the upper quartile of INPP5D intensity and the lowest quartile of INPP5D intensity. Two-way, unpaired *t*-tests. **i** Representative WB of TBS extracts from postmortem human brain, and INPP5D WT and KO iMGs for INPP5D and GAPDH. Vertical line shows INPP5D bands quantified. GAPDH portion of the blot containing iMG samples were digitally over-exposed for presentation purposes. **j**, **k** Western blot quantification of INPP5D and GAPDH in TBS postmortem brain (prefrontal cortex, PFC) brain extracts using two antibodies to INPP5D. Kruskal–Wallis test with Dunn’s multiple comparisons test. Quantification of peptides mapping to INPP5D (Q92835) in ROSMAP brain samples extracted with urea (**l–n**) or TBS (**o**) and quantified using TMT-MS, urea TMT-MS data generated and reported in^34^. For **l**, *n* = 99 AD, 121 HP-NCI, and 101 LP-NCI human subjects. For **o**, *n* = 10 AD, 8 HP-NCI, and 12 LP-NCI human subjects. Kruskal–Wallis test with Dunn’s multiple comparisons tests performed in (**l**–**o**). **p**. Schematic summarizing data presented in this figure. For all panels: ns = not significant, *p < 0.05, ***p < 0.001, ****p < 0.0001. LP = no neuropathological diagnosis of AD, HP = neuropathological diagnosis of AD, NCI = not cognitively impaired, AD = clinical and neuropathological diagnosis of AD. *n* = number of human subjects and is represented by dots in **j**, **k**, **m**, **n** For all graphs, Data are presented as mean values ± SEM. See also Supplementary Data 9–13 and Supplementary Data 2, 3 for full datasets. See also Supplementary Figs. 2–4.

We next used immunocytochemistry (ICC) to examine INPP5D levels in a cohort of human brain tissue (temporal cortex; 8 NCI, 8 AD; Supplementary Data 13). These brain samples were fixed, sectioned, and immunostained for INPP5D, IBA1, and Aß (Fig. 2a–c). INPP5D was readily detected in microglia and was dispersed throughout the cytoplasm with occasional bright puncta in the cell soma (Fig. 2a–c). Quantification of the intensity of immunostaining demonstrated overall higher levels of INPP5D staining in AD brains when compared to NCI controls (Fig. 2d, e, Supplementary Data 3). Elevated *INPP5D* transcripts also were observed in AD brain (Supplementary Fig. 2), in line with previous reports^20,36,37^. When separating microglia based on proximity to Aß plaques (“none” vs “plaque”), this elevation in INPP5D intensity in AD brain tissue is largely driven by plaque-associated microglia (Fig. 2f).

We next examined the relationship between INPP5D levels and the morphology of microglia and compared this relationship in plaque associated and non-plaque associated microglia in NCI and AD (Fig. 2g). All four categories of microglia show a strong, inverse correlation between INPP5D intensity and a quantification of ramification status (“form factor”), suggesting that less ramified microglia (putatively more activated) express lower levels of INPP5D. A steeper slope is observed for plaque-associated microglia in AD compared to the other categories, suggesting that the plaque-associated microglia in the AD brain that have the highest levels of INPP5D are more ramified (putatively less activated) than their counterparts in NCI and, conversely, that lower levels of INPP5D in AD plaque-associated microglia are more ameboid in morphology.

In brain microglia, INPP5D protein was observed to be diffusely localized in some microglia while other microglia showed intense, punctate staining, consistent with the formation of INPP5D-rich puncta. Quantification of the pattern of INPP5D distribution with each microglia revealed a shift in AD microglia to more punctate localization compared to brain microglia from individuals who were not cognitively impaired (Fig. 2h).

### Full-length aqueous-soluble INPP5D protein levels are reduced in the AD brain

To better understand the levels of active INPP5D in the human brain in the context of AD, we next examined human brain tissue (prefrontal cortex, PFC) from the ROS-MAP cohorts^30–34^ for protein expression of INPP5D by western blot and tandem mass tag-mass spectrometry (TMT-MS). We compared INPP5D levels across three categories: low pathology, not cognitively impaired (LP-NCI), high pathology, not cognitively impaired (HP-NCI) and AD. LP-NCI individuals did not have a clinical or pathological diagnosis of AD. HP-NCI had a pathological diagnosis of AD but no cognitive impairment, and “AD” had both clinical and pathological diagnoses of AD. INPP5D encodes a cytosolic protein with a predicted molecular weight in its full-length form of approximately 145 kDa. Multiple isoforms have been reported, some of which encode forms of INPP5D that do not contain the phosphatase domain and are predicted to be inactive^20,38^. Medial prefrontal cortex (mPFC) samples from a cohort of 92 individuals (Supplementary Fig. 3, Supplementary Data 9) were homogenized in tris buffered saline (TBS) to extract aqueous-soluble, cytosolic proteins and western blotting was performed to quantify full-length INPP5D. Two different INPP5D antibodies were used, C40G9 and ab45142. Although these antibodies have slightly different banding patterns, both appear to be specific to INPP5D, as demonstrated by loss of these bands in INPP5D biallelic loss-of-function iMG samples (Fig. 2i). A significant reduction in INPP5D protein levels was observed in AD and HP-NCI groups compared to LP-NCI brain tissue using both antibodies (Fig. 2j, k, Supplementary Data 10). Analyses of associations of TBS protein levels with the AD risk SNP rs10933431-C suggest a trend toward an association with reduction in levels in AD brain tissue, although the numbers for the minor allele are too low to make definitive conclusions (Supplementary Fig. 3).

While INPP5D has been described to function in the cytosol, it may be partitioned into multiple subcellular compartments. Further, in addition to Aß and tau, a multitude of proteins accumulate in insoluble aggregates in the “high pathology” elderly brain. We next obtained a second cohort of ROSMAP brain tissue (DLPFC) extracted with urea. Quantification of INPP5D in urea-solubilized human brain tissue by western blot revealed slightly reduced levels of INPP5D in the population of AD brain tissues relative to NCI individuals, with lower levels in AD relative to HP-NCI (Supplementary Figs. 3 and 4, Supplementary Data 11, Supplementary Data 2). Analyses of peptide-level data of INPP5D using TMT-MS^34^ following extraction with urea or TBS both revealed that peptides mapping to the phosphatase domain are reduced in AD while C-terminal peptides are elevated (Fig. 2l–o, Supplementary Data 12).

Taken together, these protein-level analyses of INPP5D in the postmortem brain suggest that full-length, aqueous-soluble INPP5D is reduced in the AD brain while truncated, C-terminus containing isoforms are elevated (Fig. 2p). The consequences of this complex regulation of INPP5D in AD microglia will be revisited in subsequent sections.

### Acute inhibition of INPP5D induces alterations of RNA and protein expression profiles associated with innate immune processes

**Fig. 3:**
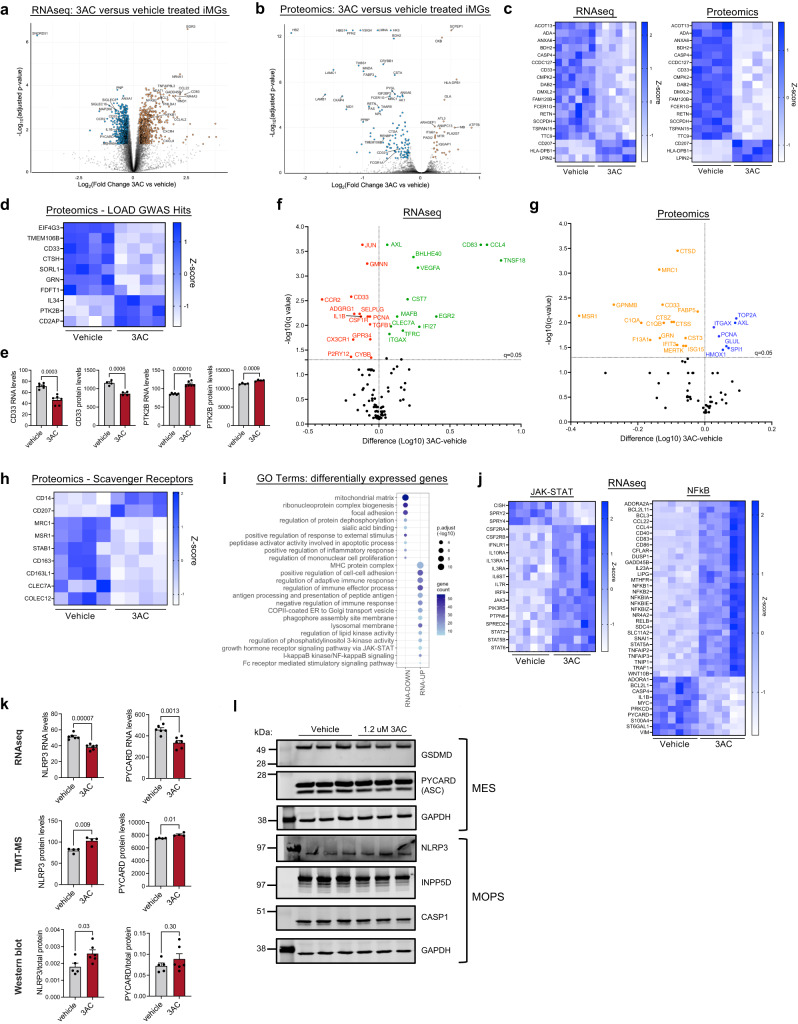
INPP5D inhibition induces changes in pathways associated with innate immunity. **a**, **b** iMGs were treated with vehicle (ethanol) or 3AC (1.25 μM) for 6 h. **a** Cells were then lysed, RNA purified, and RNAseq performed, *n* = 6 per condition. Volcano plot of DEGs comparing vehicle and 3AC treatment conditions. **b** Cells were lysed in urea and TMT-MS performed. *n* = 4 per condition. Volcano plot of DEPs; For **a**, **b** differential expression was calculated using a linear modeling with empirical Bayesian statistical analysis using the limma package in R. All *p*-values are adjusted using the Benjamini Hochberg (BH) procedure. **c** Heatmap of expression of DEGs that showed concordant differential expression at the RNA and protein level. **d** Heatmap of relative expression of DEPs encoded by LOAD GWAS candidate genes^19^ between vehicle and 3AC-treated iMGs. **e**. Relative RNA and protein levels of CD33 and PTK2B between vehicle and 3AC-treated microglia, as quantified by RNAseq and TMT-MS. Mean ± SEM. Two-sided Welch’s *t*-test. **f**, **g**. A variety of microglial subtypes previously have been defined through single-cell sequencing, with specific subtypes implicated to be altered in AD brain and model systems^49–53^. Volcano plots comparing 3AC vs vehicle treatment for genes defining these subtypes within the transcriptomic and proteomic datasets are shown; significance determined by BH FDR < 0.05. **h** Heatmap of relative protein-levels of scavenger receptors in vehicle and 3AC-treated iMGs. **i**, **j**. Enriched GO terms of biological processes for the differentially expressed genes that are elevated with 3AC treatment (geneontology.com^54^); Fisher’s exact test, Bonferroni multiple comparison’s test, adj *p*-value as shown by size of circles. **j** Heatmap of relative expression between vehicle and 3AC treatment of DEGs associated with NFκB signaling and JAK-STAT signaling. **k** Relative abundance measures of RNA or protein levels of inflammasome related components in vehicle and 3AC-treated iMGs. Mean ± SEM. Two-sided Welch’s *t*-test. **l** Representative western blot of iMGs treated with either vehicle (ethanol) or 3AC (1.25 μM) for 6 h showing protein expression of INPP5D and inflammasome related proteins: CASP1, PYCARD/ASC, GSDMD and NLRP3, as well as GAPDH. Similar WB results obtain in 3 separate differentiations. Cell lines used for experiments and full datasets for can be found in Supplementary Data 4 and 5, Supplementary Data 14. For all graphs, the number of biological replicates is represented by dots. For all panels: **p < 0.01, ***p < 0.001, ****p < 0.0001; ns = p < 0.05. See also Supplementary Figs. 5 and 6.

We next interrogated a model of decreased INPP5D activity through transcriptomic and proteomic profiling following acute inhibition. Here, we used 3-alpha-Aminocholestane (3AC), a compound previously reported to be a selective inhibitor of INPP5D^39^, which has been utilized in several studies to reduce INPP5D activity in vivo in mice and in vitro cell culture^39–47^. A recent study reported reduced INPP5D activity in primary mouse microglia treated with 3AC at a concentration (1.25 μM) well below its reported IC_50_ (10 μM)^39,45^. Here, iMG cultures were treated with this low concentration for 6 h. Cells were then collected for RNA sequencing and proteomic profiling by tandem mass tag-mass spectrometry (TMT-MS). After filtering to remove weakly detected gene-level transcripts, 12,669 genes were quantified and of these 2004 were differentially expressed between vehicle and 6 h 3AC treatment (Fig. 3a, Supplementary Data 4, Supplementary Data 14). 7866 proteins were quantified by TMT-MS, and 159 differentially expressed proteins (DEPs) were identified (Fig. 3b, Supplementary Data 5). Examination of overlap between RNA and protein-level changes revealed a set of genes that showed concordant changes in both RNA and protein datasets including *CD33*, a gene associated with LOAD through GWAS, and FCER1G, a component of Fc receptors which has been reported to biochemically interact with INPP5D^48^ (Fig. 3c, Supplementary Fig. 5). Examination of AD-GWAS associated genes revealed nine genes in addition to *CD33* which were differentially expressed at the protein level: EIF4G3, TMEM106B, CTSH, SORL1, GRN, FDFT1, IL34, PTK2B, CD2AP (Fig. 3d, e).

A variety of microglial subtypes have been defined through single-cell sequencing, with specific subtypes implicated in AD brain and model systems (for example, homeostatic, disease-associated microglia “dam”, and microglial neurodegenerative phenotype “MGnD”)^49–53^. We examined expression of genes defining these subtypes within the transcriptomic and proteomic datasets (Fig. 3f, g, Supplementary Fig. 6). We observe a protein-level decrease of lysosomal proteins CTSD and CTSS and of complement proteins C1QA and C1QB in response to INPP5D inhibition. Strong decreases in cell surface proteins such as MRC1 and MSR1 also were observed with INPP5D inhibition. Both MRC1 and MSR1 are categorized as scavenger receptors and several other scavenger receptors also showed a protein-level decrease with 3AC treatment (Fig. 3h). While expression of several of these microglia-subtype genes were altered with INPP5D inhibition, we did not observe a clear shift from one defined subtype to another with INPP5D inhibition (Supplementary Fig. 6). We next used GeneOntology analyses (geneontology.org^54^) to characterize biological processes and pathways affected by INPP5D inhibition. Pathways upregulated with 3AC included regulation of autophagy, regulation of cytokine production, and NFκB signaling (Fig. 3i, j). As NFκB signaling plays a critical role in mediating inflammatory responses, we examined the RNA and protein level changes of genes central to this pathway. Interestingly, key components of inflammasome signaling such as PYCARD (ASC), CASP1, IL1B, and NLRP3 were differentially expressed at the RNA level (Fig. 3k). Western blotting confirmed the expression of inflammasome components in iMGs at baseline and with 3AC treatment, but contrary to RNA levels showed elevations in ASC and NLRP3 protein levels with 3AC, changes also observed through TMT-MS (Fig. 3k, l). These changes following INPP5D inhibition implicate responses in innate immune signaling pathways and suggest a reduction in INPP5D activity may lead to inflammasome activation that also induces a negative feedback loop to repress transcription of inflammasome components.

### INPP5D inhibition results in inflammasome activation

**Fig. 4:**
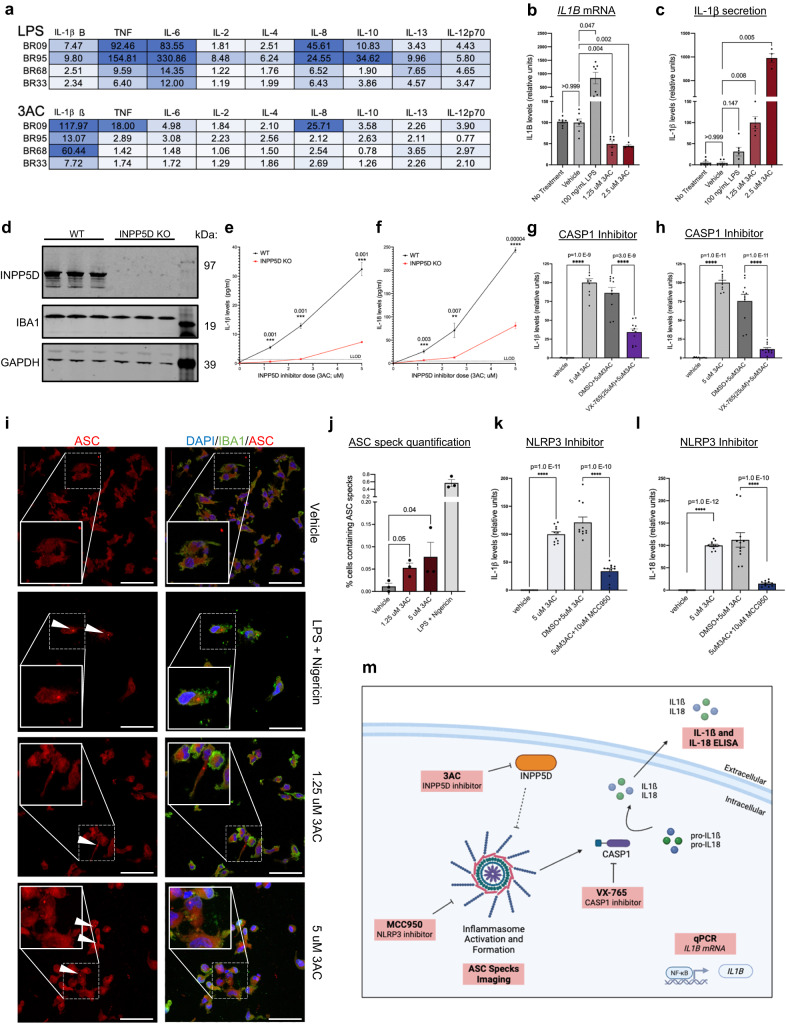
Acute INPP5D inhibition results in inflammasome activation and an increase in the secretion of IL-1ß and IL-18 in iMG cultures. **a** Fold change of secreted cytokines from iMGs treated with LPS (100 ng/mL) or 3AC (1.25 μM) for 6 h across iMGs derived from four iPSC lines, assayed by ELISA. Fold change was calculated compared to vehicle-treated cells. **b** Levels of *IL1B* following 6 h treatment with vehicle, LPS (100 ng/mL), or 3AC (1.25 μM, 2.5 μM) treatment as measured by quantitative real-time PCR (qPCR). Data are normalized to *GAPDH* expression then values were normalized to vehicle treatment within each experiment. *n* = 3 differentiations with well-level data shown as dots. One-way ANOVA with Dunnett’s T3 multiple comparisons test. **c** Secreted levels of IL-1ß measured for the same experiments as (**b**) following 6 h treatment as measured by ELISA. IL-1ß levels were normalized to 1.25 μM 3AC-treated conditions within each experiment. *n* = 3 differentiations with well-level data shown as dots. **d** Representative western blot of protein expression in iMGs with biallelic loss-of-function mutations generated with CRISPR targeting. Loss of INPP5D protein observed repeatedly over 3 differentiations. **e**, **f** WT INPP5D iMGs and KO INPP5D iMGs were treated with 3AC for 6 h and the levels of IL-1ß and IL-18 were measured by ELISA. Multiple two-sided *t*-tests, Two-stage step-up (Benjamini, Krieger, and Yekutieli), **q < 0.01; ***q < 0.005; ****q < 0.001; LLOD= lower limit of detection. *n* = 3 biological replicates. **g**, **h** Treatment of iMGs with either vehicle or 3AC (5 μM) with either VX-765 (25 μM) or its vehicle (DMSO). Levels of secreted IL-1ß and IL-18 measured via ELISA following treatments and normalized to 3AC-treated samples in each experiment. One-way ANOVA with Sidak’s multiple comparison test. 3 differentiations, *n* = 10 per condition. **i**, **j** iMGs were treated with either vehicle, primed with LPS (100 ng/mL) for 3 h and then treated with nigericin (10 μM) for 1 h, or treated with 3AC (1.25 μM or 5 μM) for 2 h. Cells were immunostained for ASC and IBA1, DNA is stained with DAPI (i) and imaged using confocal microscopy. Scale bars = 100 μm. **j** Quantification of ASC specks. Number of cells analyzed: *n* = 226 (vehicle); 151 (1.25 μM 3AC); 173 (5.0 μM 3AC) over three experiments and 10 images per condition. Kruskal–Wallis test to determine significance. **k**, **l** Treatment of iMGs with either vehicle or 3AC (5 μM) with either MCC950 (10 μM) or its vehicle (DMSO). Levels of secreted IL-1ß and IL-18 in the media were measured and normalized to 3AC-treated samples in each experiment. One-way ANOVA with Sidak’s multiple comparison test. *n* = 4 differentiations with well-level data shown as dots. **m** A summary figure of experiments performed to interrogate inflammasome activation, created using BioRender.com. For all graphs, data are presented as mean values ± SEM. For **b**, **c**, **g**, **h**, **j**, **l**: ns = not significant, *p < 0.05, **p < 0.01, ***p < 0.001, ****p < 0.0001. Cell lines used for experiments are detailed in Supplementary Data 8. See also Supplementary Figs. 7 and 8.

To test whether INPP5D inhibition impacts profiles of secreted cytokines, iMGs were treated with 3AC (1.25 μM) and cytokine response assayed. Conditioned media were collected following a 6 h treatment and 9 proinflammatory cytokines were quantified (MesoScale Discoveries, IL-1ß, IL-2, IL-4, IL-6, IL-8, IL-10, IL-12p70, IL-13, and TNF). Levels of cytokines were normalized to vehicle-treated cells and benchmarked against effects of treatment with lipopolysaccharide (LPS) (Fig. 4a). LPS-treated iMGs resulted in dramatic (up to 300-fold) increases in TNF, IL-6, IL-8, IL-10, and IL-13. With 3AC treatment, several cytokines were elevated, but IL-1ß showed the highest fold change in secretion that was consistently observed across multiple iMG differentiations and multiple genetic backgrounds (Fig. 4a, Supplementary Fig. 7). The increase in IL-1ß was even greater than observed with LPS across all genetic backgrounds.

We next measured *IL1B* mRNA levels following treatment with 3AC (1.25 μM, 6 h) to determine whether INPP5D activity affects *IL1B* transcription. iMGs were treated with LPS (100 ng/mL, 6 h) in parallel as a positive control for upregulation of *IL1B* mRNA, and *IL1B* mRNA measured by quantitative RT-PCR (qPCR). Surprisingly, *IL1B* mRNA levels did not increase following 3AC treatment. Rather, 3AC treatment induced a *reduction* in *IL1B* mRNA while increasing IL-1ß protein secretion (Fig. 4b, c), signifying that INPP5D inhibition induces IL-1ß post-translational processing and secretion while inducing a negative feedback loop to repress RNA expression of *IL1B*. The inflammasome, an innate immune sensing multimeric oligomeric complex, contributes to the maturation of IL-1ß and IL-18 primarily through caspase-1 cleavage and activation. As IL-18 is also cleaved and activated in a similar manner as IL-1ß, we measured the secretion of IL-18 in the conditioned media of 3AC-treated iMGs by ELISA. Like IL-1ß, INPP5D inhibition induced secretion of IL-18 across iMGs of seven different genetic backgrounds (Supplementary Fig. 7). Although not powered to conclusively determine if INPP5D levels are associated with IL-18 and IL-1ß secretion, IL-18 levels were elevated with lower INPP5D levels in this limited iMG cohort (Supplementary Fig. 7). 3AC treatment of other iPSC-derived cell types that do not express INPP5D (iNs and iAs) did not yield an increase in IL-1ß or IL-18 secretion, supporting the specificity of the effect of 3AC on INPP5D at the relatively low concentrations used here (Supplementary Fig. 7). Likewise, treatment of CRISPR-generated INPP5D KO iMGs with 3AC (Fig. 4d–f) did not result in detectable levels of IL-1ß and IL-18 secretion at 1.25 μM, and a dramatically reduced response even up to 5 μM, demonstrating that the majority of increased IL-1ß and IL-18 secretion is likely due to the reduction of INPP5D activity at these concentrations.

Following its formation, the inflammasome cleaves pro-caspase-1 to generate the active form of caspase-1. Western blotting of iMG lysates revealed an increase in cleaved caspase-1 with acute (2–3 h) INPP5D inhibition with 3AC (Supplementary Fig. 8a). We reasoned that if active caspase-1 is involved in the INPP5D-inhibition-induced increase of IL-1ß and IL-18 secretion, then blocking caspase-1 should prevent the induction of IL-1ß and IL-18 secretion with 3AC. iMGs were treated with either vehicle (ethanol) or 3AC (5 μM) along with either vehicle (DMSO) or caspase-1 inhibitor VX-765 (25 μM) for 6 h. Conditioned media were collected and levels of secreted IL-1ß and IL-18 measured by ELISA. Indeed, co-treatment with VX-765 prevented the INPP5D-inhibitor induced elevation in IL-1ß and IL-18 (Fig. 4g, h). Treatment with a second caspase-1 inhibitor, Ac-YVAD-cmk, also prevented the 3AC-induced elevation IL-1ß and IL-18 secretion (Supplementary Fig. 8b, c). These results suggest that active caspase-1 is required for INPP5D-mediated effects on IL-1ß and IL-18 secretion.

Immunostaining for the inflammasome adapter protein, ASC (PYCARD) allows for visualization of inflammasome formation as the oligomerization of ASC within the inflammasome will form a “speck” within the cell^55^. As a positive control, iMGs were first primed with LPS (100 ng/mL) for 3 h and then treated with an inflammasome activator, nigericin (10 μM), for 1 h prior to fixing and immunostaining for ASC. In parallel, iMGs were treated with 3AC (1.25 μM, 5 μM) for 4 h and fixed and immunostained for ASC. ASC specks of about 1 μm in size, consistent with what is reported in literature^55^, were detected in a subset of the iMGs treated with LPS and nigericin and in the iMGs treated with 3AC. With 5 μM 3AC, significantly more ASC specks were observed compared to the vehicle-treated conditions, but almost 10-fold fewer than observed in iMGs treated with LPS and nigericin (Fig. 4j–i).

Next, we utilized an NLRP3 inhibitor, MCC950, to inhibit inflammasome formation^56^. MCC950 binds to NLRP3 and prevents a conformation change necessary for inflammasome formation, thus preventing inflammasome activation^57^. iMGs were treated with either vehicle (ethanol) or 3AC (5 μM) along with either vehicle (DMSO) or MCC950 (10 μM) for 6 h and then conditioned media were collected to measure levels of secreted IL-1ß and IL-18 (Fig. 4k, l). Inhibition of NLRP3-inflammasome formation prevented the effects of 3AC on IL-1ß and IL-18 secretion, signifying that inflammasome formation occurs downstream of INPP5D inhibition. Taken together, the visualization of ASC specks, CASP1 inhibitor treatments, and MCC950 treatment experiments support the hypothesis that INPP5D phosphatase activity suppresses inflammasome activation in iPSC-derived microglia (Fig. 4m).

### Chronic, genetically induced reduction of INPP5D induces protein-level changes in immune response proteins and increased secretion of IL-1ß and IL-18

**Fig. 5:**
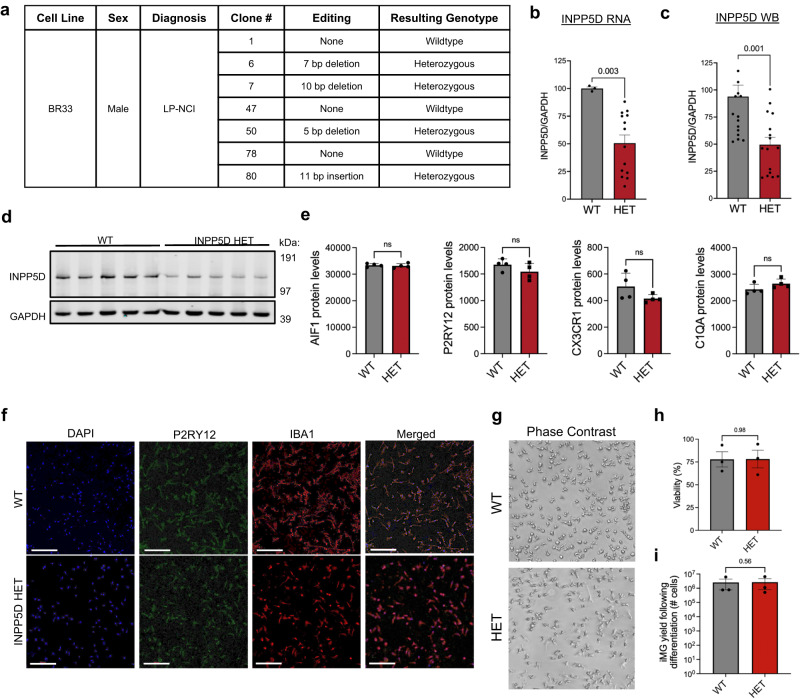
Generation of INPP5D heterozygous iMGs using CRISPR targeting. **a** Table of CRISPR-Cas9 generated INPP5D heterozygous (HET) and monoclonally selected wild-type (WT) lines. **b** INPP5D RNA levels measured by qPCR, normalized to GAPDH levels. Two-tailed Mann–Whitney test. *n* = 3 differentiations with replicate wells as shown by dots. **c** Western blot quantification of INPP5D protein levels in INPP5D WT and HET iMGs normalized to GAPDH levels; Two-tailed Mann–Whitney test. *n* = 3 differentiations with replicate wells as shown by dots. **d** Representative western blot (of three differentiations) of INPP5D, GAPDH, and IBA1 protein levels in INPP5D WT and HET iMGs. **e** INPP5D WT and HET iMGs were lysed in urea and TMT-MS performed, *n* = 4 WT, *n* = 4 HET. Relative protein levels of AIF1, P2RY12, CX3CR1, C1QA; Unpaired two-sided *t*-test. **f** Representative images (of three independent differentiations) of INPP5D WT and HET cells immunostained for IBA1 and P2RY12. DNA is stained with DAPI. Imaged using confocal microscopy. Scale bars = 200 μm. **g** Phase contrast images of INPP5D WT and HET iMGs. **h** Viability was determined by measuring the level of LDH secretion normalized to total measure of LDH following cell lysis. *n* = 3. Two-sided Mann–Whitney test: ns = no significance (*p* > 0.05). **i**. Quantif**i**cation of the cell yield following differentiation to iMG fate comparing INPP5D WT or HET lines, 2-sided paired *t*-test, *n* = 3. For **b**, **c**, **e:** ns not significant *p* > 0.05, **p < 0.01. For all graphs, data are presented as mean values ± SEM.

The experiments outlined above suggest that acute reduction in INPP5D phosphatase activity results in inflammasome activation. Reduction in full-length TBS-soluble INPP5D protein levels in the brain would result in chronic reduction of INPP5D activity which may have different outcomes compared to an acute, efficient inhibition of INPP5D phosphatase activity. To interrogate the consequences of a chronic reduction of INPP5D activity, CRISPR-Cas9 was used to introduce loss-of-function mutations at the INPP5D locus in a single allele. The human iPSC line chosen was derived from a cognitively normal individual with minimal (lowest quartile within age 65+ cohort) neuritic plaque and tangle burden in their brain at death at age 90+ years (BR33^26^). Heterozygous loss-of-function mutations (“HET”) were identified in several monoclonal lines, and wild-type monoclonal lines also were isolated that were subjected to the same process. Figure 5a outlines the mutations introduced and clones isolated (see also Supplementary Data 15). In microglia derived from each of the heterozygous clones, both INPP5D mRNA and protein levels were reduced by approximately 50% (Fig. 5b–d).

**Fig. 6:**
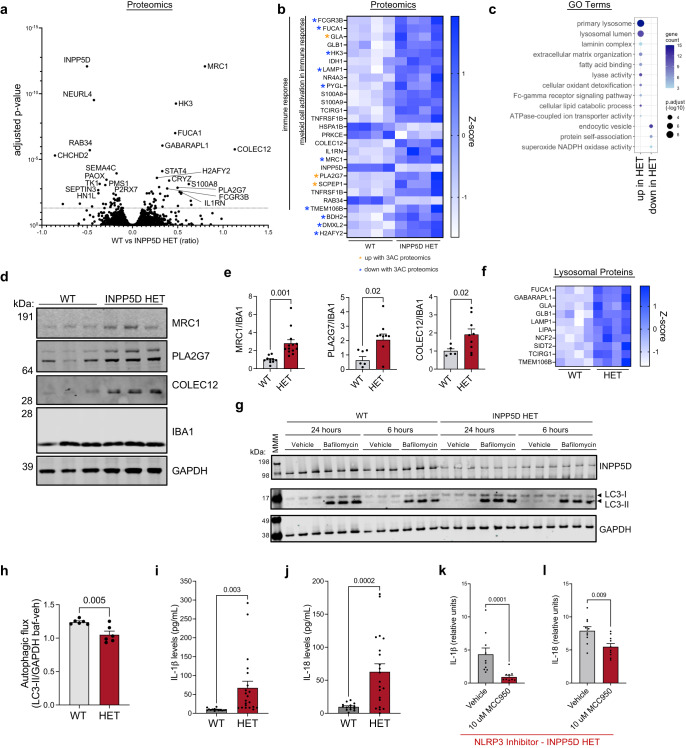
Chronic reduction in INPP5D levels results in inflammasome activation and reduction in autophagic flux. **a–c** Volcano plot (**a**) of adjusted *p*-values showing 71 DEPs from the 7868 quantified via TMT-MS (two-way *t*-test with Benjamini–Hochberg multiple comparisons test; FDR < 0.05. Heatmap (**b**) of relative expression levels of proteins involved in immune signaling that are differentially up and downregulated in INPP5D WT vs HET iMGs. Asterisks indicate proteins that also were differentially up or downregulated (*q* < 0.05) with 3AC treatment (Fig. 3). GO analysis (**c**) of DEPs; Fisher’s exact test, Bonferroni multiple comparison’s test, adj *p*-value as shown by size of circles. **d**, **e** Representative western blot and quantification across three differentiations of MRC1, PLA2G7, COLEC12, IBA1, and GAPDH in INPP5D WT and HET iMGs. *n* = 3 independent experiments, mean ± SEM; two-sided *t*-test. **f** Heatmap of TMT-MS data (z-score) for lysosomal DEPs in INPP5D HET vs WT iMGs. **g** Representative western blot of INPP5D iMGs treated with either vehicle (DMSO) or bafilomycin (baf, 100 nM) for either 24 or 6 h. Western blots are probed for INPP5D and LC3 with GAPDH as a loading control. **h** Quantification of autophagic flux (baf - veh of LC3-II/GAPDH) in iMGs with treatment of 24 h of baf. *n* = 6 wells vehicle, *n* = 6 wells of 6 h baf. Mean ± SEM, two-sided *t*-test with Welch’s correction. **i**, **j** Secreted IL-1ß and IL-18 measured by ELISA of media collected from INPP5D HET and WT iMGs. Media were concentrated tenfold in order to be above the limit of detection and normalized to WT mean. *n* = 14 wells WT, *n* = 21 wells HET. Mean ± SEM. Two-sided Mann–Whitney test. **k**, **l** Secreted IL-1ß and IL-18 measured by ELISA of media collected from INPP5D HET iMGs treated with either vehicle (DMSO) or MCC950 (10 μM) for 24 h. Media were concentrated tenfold for detection. *n* = 10 wells vehicle, *n* = 10 wells MCC950-treated. Mean ± SEM. Mann–Whitney test. For **e**, **h**–**l** *p < 0.05, **p < 0.01, ***p < 0.001, ****p < 0.0001. See also Supplementary Fig. 9, Supplementary Data 4 and 5 for full datasets.

We next examined the consequences of chronic loss-of-function of one allele of INPP5D on protein profiles. Expression of the microglial proteins AIF1, P2RY12, CX3CR1, and C1QA were not significantly changed between WT and HET iMGs, demonstrating that one less functional copy of INPP5D did not grossly alter the differentiation process (Fig. 5e) nor did it significantly affect immunostaining for the microglial markers IBA1 and P2RY12, morphological features, viability, or differentiation yield (Fig. 5f–i). Differential protein analysis between WT and INPP5D HET iMGs revealed 71 differentially expressed proteins—38 proteins were significantly increased in the INPP5D HET iMGs and 33 proteins were significantly decreased (Fig. 6a, Supplementary Fig. 9). There was no significant change in expression of functionally related genes such as *INPPL1* in the INPP5D HET, demonstrating that there is no obvious compensatory upregulation of similar phosphatases with a reduction in INPP5D levels (Supplementary Fig. 9).

Downregulated proteins in INPP5D HET iMGs were enriched in pathways involving protein folding and stability such as TTC9, PDIA5, USOA1B and IRF2BPL, as well as proteins previously implicated in microglial dysfunction such as SEPTIN3 and P2RX7, the latter of which has been linked to inflammasome activation^58,59^. Proteins upregulated in INPP5D HET iMGs include many falling under the GO term of “myeloid cell activation in immune response” such as PYGL, TCIRG1, GLA, FCGR3B, TNFRSF1B, HK3, S100A9, S100A8, GLB1, IDH1, FUCA1, LAMP1, NR4A3. Additionally, GSEA showed enrichment in pathways relevant to the lysosome (Fig. 6b, c). Through western blot analyses, we also confirmed the significant upregulation of MRC1, PLA2G7 and COLEC12 in the INPP5D HET iMGs (Fig. 6d, e). Intriguingly, MRC1 and COLEC12 were two genes defining cluster 13 (Fig.1f–i), which shows a reduction in INPP5D in human brain. DEPs enriched in lysosomal proteins included CYBRD1, CTSA, SIDT2, TMEM106B, FUCA1, TCIRG1, GLB1, NCF2, GLA, LAMP1, and LIPA (Fig. 6f). We confirmed that this dysregulation had a functional impact by measuring autophagic flux: INPP5D WT and HET iMGs were treated with either vehicle (DMSO) or bafilomycin (100 nM) for 24 or 6 h. A significant reduction in autophagic flux was observed in iMGs with reduced INPP5D expression (Fig. 6g, h).

Upregulation of proteins which are implicated as negative regulators of innate immune activation (such as IL1RN^60^) suggest there may be a negative feedback mechanism with a chronic reduction in INPP5D activity. Comparison of the DEPs between acute (vehicle vs 3AC) and chronic (WT vs INPP5D HET) reduction revealed 14 DEPs shared between the two analyses (Supplementary Fig. 9). Intriguingly, 10 of the proteins that were downregulated with 3AC treatment were upregulated in the INPP5D HET (BDH2, DMXL2, FCGR3B, FUCA1, H2AFY2, HK3, LAMP1, MRC1, PYGL, and TMEM106B), a number higher than would be expected by chance (Chi square test, *p* < 1 × 10^−15^; Supplementary Fig. 9). Thus, reduction in INPP5D activity may result in inflammasome activation and induction of a negative feedback mechanism that results in lowering of *IL1B* RNA levels (as in Fig. 4b).

Despite this potential feedback mechanism, a chronic decrease of INPP5D levels resulted in an elevation in extracellular secretion of IL-1ß and IL-18 (Fig. 6i, j). It is worth noting that the conditioned media needed to be concentrated 10-fold in order for these cytokines to be within detection range. This is in contrast to acute INPP5D inhibition, which results in readily detectable levels without media concentration. To determine whether this increase was the result of inflammasome activation, we treated INPP5D HET iMGs with either vehicle (DMSO) or NLRP3 inhibitor, MCC950 (10 μM), for 24 h. Following treatment, a significant decrease in IL-1ß and IL-18 secretion with MCC950 treatment was observed (Fig. 6k, l). Taken together, these data demonstrate that a chronic reduction of INPP5D results in reduction in autophagic flux, sub-lytic levels of inflammasome activation and protein-level changes in immune response genes.

### Evidence for inflammasome activation with reduced INPP5D levels in AD human brain tissue

Based upon these in vitro data, we hypothesize that reduction of INPP5D activity in the human brain results in inflammasome activation. Results presented in Fig. 2 suggest that there are different pools of INPP5D in the brain that are differentially detectable using different methods. However, none of the described methods relays whether and how the active pool of INPP5D is altered in AD brain. To address this, we first examined the hypothesized connection between INPP5D levels and inflammasome activation within the AD brain. We then used an unbiased approach (RNAseq) to examine whether pathways altered in AD microglia in the brain were concordant with INPP5D gain- or loss-of-function microglia.

**Fig. 7:**
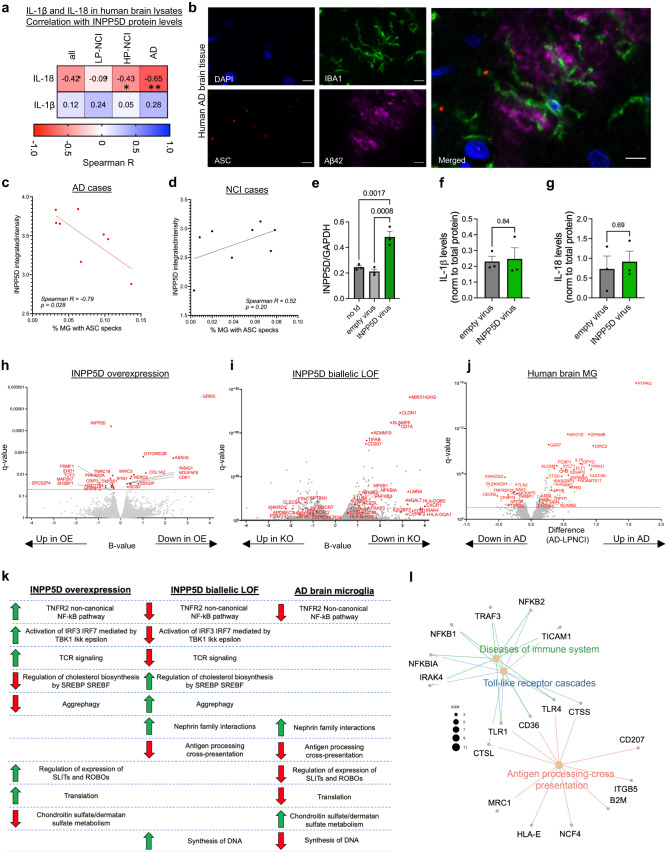
Evidence linking INPP5D levels and inflammasome activation in the human brain. **a** IL-1ß and IL-18 levels were measured from human TBS brain lysates and normalized to total protein. Spearman correlation coefficients were calculated between IL-1ß and IL-18 levels (normalized to total protein) and INPP5D/GAPDH levels (AD, HP-NCI, LP-NCI, or all). **b–d** Human brain tissues (DLPFC, BA9; 8 NCI, 8 AD) were fixed, sectioned, and immunostained for ASC, IBA1 and Aβ. These sections were derived from the same batch as used in Fig. 2h–m to quantify INPP5D levels. Example immunostaining of human brain sections (6 microns) for IBA1, ASC, and Aβ is shown in (**b**). DNA is stained with DAPI. Imaged using confocal microscopy. Images are representative of data obtained from brain sections from 16 individuals. Scale bars = 10 μm. Spearman correlation coefficients were calculated comparing the percent of microglia containing ASC specks and the average intensity of INPP5D immunostaining by human subject, analyses performed separately on AD and NCI subjects (**c**, **d**). **e** WT iMGs were transduced with either an empty lentivirus plus VSV-G virus-like particles (VLPs), a lentivirus encoding an INPP5D overexpression cassette (OE) plus VLP, or not transduced, and 72 h later the cells were lysed, RNA purified and the levels of *INPP5D* RNA quantified by qPCR with normalization to *GAPDH*. *n* = 4 biologically independent samples; data are presented as mean values ± SEM. One-way ANOVA with Dunnett’s multiple comparisons test; **p < 0.01, ***p < 0.001. **f**, **g** From the same cells, levels of IL-1ß and IL-18 were measured in conditioned media following 72 h of viral transduction, data are presented as mean values ± SEM. *n* = 4 biologically independent samples, two-sided *t*-test**. h** RNAseq was performed on samples cultured in parallel, volcano plot of DEGs observed with INPP5D overexpression is shown. RNAseq also was performed on iMGs derived from iPSC lines with biallelic INPP5D loss-of-function mutations and their isogenic WT controls. Volcano plot of DEGs is shown in **i**. Pseudobulk snRNAseq data of microglia^36,37^ was analyzed to identify DEGs between microglia from AD and NCI brain tissue. These data were generated from brain tissue (DLPFC, BA9) from 131 AD and 162 NCI ROSMAP participants. Volcano plot comparing AD to NCI brain tissue is shown in (**j**). GSEA was performed using the REACTOME database from DEG analysis performed in (**h–j**). **k** Table of pathways that were enriched in any two of the three comparisons in (**h–j**). See Supplementary Data 16–19 and Supplementary Data 6,7 for complete DEG and GSEA results. **l** Only one pathway was enriched in all three datasets. This pathway relates to NFκB signaling and shown is the gene concept network of leading-edge genes in the INPP5D iMG loss-of-function comparison.

To examine inflammasome activation, levels of IL-1ß and IL-18 were quantified in brain lysates by ELISA and compared to levels of INPP5D in the same TBS extracts as measured by western blot (Fig. 2c–e; Fig. 7a). IL-18 levels were significantly associated with INPP5D levels in the TBS brain fractions, with lower INPP5D levels associating with elevated IL-18 levels (Fig. 7a, Supplementary Data 16). This relationship was found to be strongest in the AD brain samples when subdivided by diagnosis. IL-1ß was not associated with INPP5D levels, perhaps due to the contribution of astrocytes and other cell types to its levels within the brain, or to differences in the regulation of IL-18 and IL-1ß secretion. Next, immunostaining of human brain tissue was performed to evaluate the potential relationship between INPP5D expression and ASC speck formation within microglia in the aged brain (Fig. 7b). In the AD brain, lower INPP5D levels were associated with a higher percentage of microglia with ASC specks (Fig. 7c, Supplementary Data 16). This association is not observed across NCI cases, which overall have fewer microglia with ASC specks (Fig. 7d). Together, these data support the hypothesis that lower INPP5D in AD brain microglia is associated with inflammasome activation.

We next overexpressed INPP5D in iMGs utilizing VSV-G virus-like particles (VLPs)^61^ to deliver lentiviruses encoding an INPP5D overexpression (OE) expression cassette, which resulted in a fifty percent increase in INPP5D levels (“OE”) (Fig. 7e). This increase in INPP5D levels did not induce a change in secreted IL-1ß or IL-18 levels (Fig. 7f–g). RNA sequencing was performed on both INPP5D overexpression iMGs as well as in CRISPR-generated INPP5D biallelic loss-of-function (“KO”) and WT iMGs (Fig. 7h, i). Comparison of INPP5D KO iMGs to isogenic WT control iMGs yielded 3,710 DEGs (*q*-value < 0.05), while comparison of INPP5D OE iMGs to control-transfected iMGs yielded 27 DEGs (q-value < 0.05; Supplementary Data 6, 7, Supplementary Data 17, 18). In parallel, we used single nucleus RNAseq data available on the AMP-AD Knowledge Portal from human brain tissue to compare pseudobulk RNAseq data from AD microglia to LP-NCI microglia^36,37^. This analysis yielded 260 DEGs (*q* < 0.05; Fig. 7j, Supplementary Data 19). GSEA analyses were performed using the REACTOME database, and pathways were identified that were enriched in at least two of the three comparisons (p < 0.05; Supplementary Data 20). Multiple pathways relevant to inflammasome signaling were altered with INPP5D loss-of-function and are aligned with patterns observed in AD brain microglia (compared to LP-NCI brain microglia) while pathways altered with INPP5D overexpression are more similar to those in LP-NCI brain microglia (compared to AD) (Fig. 7k). Only one pathway was significantly associated in all three comparisons: genes in the TNFR2 non-canonical NFκB pathway are downregulated at the RNA level in microglia in the postmortem AD brain and are also downregulated in iMGs with INPP5D loss-of-function (Fig. 7k); genes associated with this pathway were upregulated with INPP5D overexpression (Fig. 7k). Similarly, RNA expression of genes relating to “Antigen processing cross presentation” were downregulated in both AD brain microglia and in iMGs with INPP5D loss-of-function. Gene-concept networks of leading-edge genes driving enrichment in INPP5D KO vs WT comparisons include *NFκB1, NFκB2, TLRs*, cathepsins and *MRC1* (Fig. 7l). Taken together, these data demonstrate that the INPP5D loss-of-function iMGs more closely resemble the microglia in the AD brain compared to microglia with elevation of INPP5D protein levels.

### Loss of one copy of INPP5D in microglia has non-autonomous effects on gene expression profiles of neurons

**Fig. 8:**
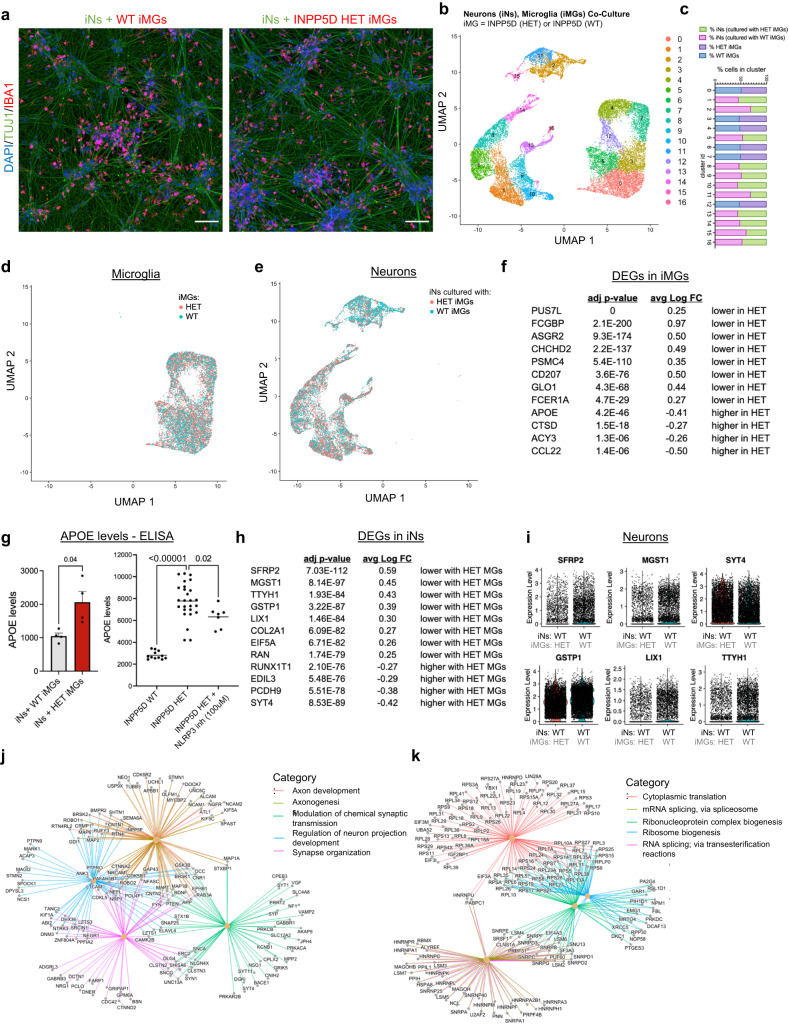
Loss of one copy of INPP5D in microglia has functional consequences on gene expression profiles of co-cultured neurons. **a** Co-cultures of iNs with either INPP5D WT or HET iMGs were fixed and immunostained for TUJ1 and INPP5D. Representative images are shown for at least three independent experiments. DNA is stained with DAPI. Imaged using confocal microscopy. Scale bars = 100 μm. **b** Clusters generated from single cell sequencing of neuron and microglia co-cultures. **c** Percent composition of each cluster based upon genotype and cell type is shown. **d**, **e** Microglial and neuronal clusters were defined by marker expression. **f** Top DEGs between cell groups were identified using the Wilcoxon Rank Sum test (2-sided) with a multiple comparisons adjusted (FDR) *p*-value cutoff of 0.05, and LogFC threshold of 0.25. **g** APOE levels in the media (as measured by ELISA) of iN and iMG co-cultures (left) or of INPP5D WT or HET iMGs, with treatment of het iMGs with NLRP3 inhibitor for 24 h (right). *n* = 4 biologically independent samples (left graph) and *n* = 12, 26, 7 (left to right) biologically independent samples (right graph); data are presented as mean values ± SEM. Two-tailed paired *t*-test (left graph) and Brown-Forsythe and Welch’s ANOVA with Dunnett’s multiple comparisons test (right graph). **h** DEGs were identified between cell groups using the Wilcoxon Rank Sum test (two-sided) with a multiple comparisons adjusted (FDR) *p*-value cutoff of 0.05, and LogFC threshold of 0.25. Top DEGs are shown in (**h**. **i**). Scatter plots of top six DEGs in iNs co-cultured with WT or HET iMGs. **j**, **k** GSEA was performed using the full dataset of iN gene expression. Gene-concept networks of leading-edge genes for the top pathways identified through GSEA are shown. Upregulated pathways in iNs cultured with HET iMGs were related to synaptic function (**j**) and those downregulated with co-culture with HET iMGs were associated with mRNA splicing and ribonucleoprotein complexes. See also Supplementary Figs. 10–12 and Supplementary Data 21–24 for full datasets. For **g**: *p < 0.05, ****p < 0.0001.

The salient feature of AD that results in cognitive decline is the loss of synapses and neuronal death. Thus, it is important to examine whether dysfunction of microglial risk genes affects neuronal biology. We provide evidence that acute and chronic reduction of INPP5D activity in microglia result in inflammasome activation and secretion of IL-1β and IL-18. Next, we aimed to examine whether loss of one copy of INPP5D effects neurons by co-culturing INPP5D WT and HET iMGs with iNs generated from INPP5D WT iPSC lines. The co-cultures were maintained for 48 h prior to dissociation for single cell (sc)RNAseq to characterize transcriptomic changes in both the iNs and the iMGs. Parallel wells were plated for fixation and immunostaining to examine the morphology of the cells in culture. Immunostaining for TUJ1 to label neurites and IBA1 to label microglia revealed that iMGs were well dispersed throughout the cultures under both conditions (Fig. 8a).

Through single-cell analysis, 24,717 cells (11,201 iMGs and 13,517 iNs) were identified with an average number of mapped genes detected per cell of 5836 for iMGs and 6596 for iNs, and average total UMIs (unique molecular identifiers; unique RNA strands captured) per cell of 37,027 (iMGs) and 37,112 (iNs). UMAP clustering revealed 17 clusters with a minimum of 10 cells per cluster (Fig. 8b, c, Supplementary Figs. 10, 11). There were no obvious shifts in cluster membership between either WT and HET iMGs (Fig. 8c, d) or between iNs based on the genotype of the co-cultured iMGs (Fig. 8c, e).

First, differential gene expression was examined between WT and HET iMGs, and the top 12 DEGs (defined by adjusted *p*-value) are shown (Fig. 8f, see Supplementary Data 21, 22). These DEGs suggest that cellular processes may be impacted such as mitochondrial function (*CHCHD2*), proteasome capacity and protein degradation (*PSMC4*), and reactive oxygen species metabolism (*GLO1*)^62,63^. Additionally, *ASGR2, CD207, and FCER1A* were reduced and all three are cell surface receptors which provides additional evidence for dysregulation of scavenger receptors and related receptor signaling in iMGs following a decrease in INPP5D activity and expression. Cathepsin D (*CTSD*) also was elevated in HET iMGs, consistent with perturbed lysosomal function.

Intriguingly, *APOE* was one of the strongest upregulated genes in INPP5D HET iMGs (Fig. 8f). This elevation was confirmed at the protein level by measuring APOE in the conditioned media of iMG-iN co-cultures (Fig. 8g). Further, the elevation in APOE levels in INPP5D WT and HET iMGs also was observed in INPP5D HET iMGs cultured alone, and this increase can be partially rescued with inflammasome inhibition (Fig. 8g).

Finally, we examined whether loss of one copy of INPP5D in microglia has a measurable consequence on neurons. Indeed, 109 DEGs were identified in neurons co-cultured with HET versus WT iMGs (Fig. 8h, i–k; Supplementary Data 21, 23). The strongest DEGs downregulated in iNs exposed to HET iMGs were *SFRP2*, *MGST1*, and *TTYH1* while the strongest upregulated genes were *SYT4*, *PCDH9*, and *EDIL3* (Fig. 8h, i). GSEA following DEG analysis of iN data revealed several pathways significantly upregulated with co-culture with INPP5D HET iMGs related to synaptic function (Supplementary Fig. 12, Fig. 8j). Downregulated pathways were related to RNA splicing and ribonucleoprotein complexes (Supplementary Fig. 12, Fig. 8k). Taken together, these results demonstrate that loss of just one copy of INPP5D in iMGs is sufficient to induce transcriptional consequences in neurons.

## Discussion

A key question surrounds whether INPP5D is a feasible target for therapeutic development in AD. Selective agonists and antagonists have been developed for INPP5D^64–68^ but the biology of INPP5D in the AD brain is complex and it is not immediately apparent whether INPP5D activity should be enhanced or inhibited for therapeutic benefit. The data presented here provide the deepest investigation to date into the state of INPP5D in the AD brain. These data may appear confusing at first glance: (1) bulk RNA levels of *INPP5D* are elevated in the AD brain (Supplementary Fig. 2a); (2) intensity of immunostaining signal for INPP5D in microglia is elevated in plaque-associated microglia in the brain (Fig. 2d, e); and (3) full-length aqueous-soluble INPP5D protein levels are reduced in the AD brain (Fig. 2j, k). A recent study identified significant expression of isoforms of *INPP5D* in the brain that encode truncated protein that lack the phosphatase domain^20^. This finding may explain the seemingly conflicting results presented herein regarding protein levels of INPP5D in the AD brain: *full-length* aqueous-soluble INPP5D was dramatically reduced in the AD brain, and the elevation in INPP5D observed by immunostaining may reflect the presence of truncated and functionally inactive INPP5D protein generated from the isoforms lacking the phosphatase domain. In accord, analyses of peptide level mass spectrometry data reveal a reduction in peptides in the phosphatase domain and an elevation in peptides mapping to the C-terminus in AD brain tissue (Fig. 2). Available antibodies to INPP5D recognize epitopes C-terminal to the phosphatase domain and would recognize these truncated variants. Immunostaining data presented here reveal that INPP5D protein is redistributed in AD brain microglia from a diffuse to punctate pattern. Thus, there appear to be multiple pools of INPP5D in the AD brain. Perhaps most telling with respect to function is that INPP5D LOF iMGs more closely resemble microglia in the AD brain with respect to immune signaling alterations, supporting the hypothesis that INPP5D function is reduced in AD microglia and that inhibition of INPP5D is likely to increase rather than decrease inflammatory responses in microglia (Fig. 7k). However, caution is warranted, and further studies are necessary to determine whether INPP5D agonism and/or antagonism may prevent or alleviate the synapse loss and cognitive decline observed in AD.

It is worth noting that alternative interpretations of the data may be illuminated and supported by future studies using additional approaches. For example, further experimentation is necessary to determine whether INPP5D exists in aqueous-insoluble aggregates and/or phase-separated biomolecular condensates in microglia, and whether or not the subcellular localization of INPP5D is associated with its enzymatic activity. Further, INPP5D can act as a negative regulator of TREM2 signaling in some contexts. Thus, reduction of INPP5D activity through inhibitor treatment or reduction in copy number may result in an elevation in TREM2 signaling in microglia. Further studies are warranted to determine whether INPP5D phenotypes observed herein are mediated by alterations in TREM2 signaling.

We recognize the limitations of the current study. In vitro studies are reductionist in nature, with reduced complexity regarding cell type representation compared to the endogenous environment of the brain. We recognize that in our human iMG system, the isolation of microglia away from other cell types may result in an activated state which in turn can affect expression profiles. Thus, our in vitro monoculture system of iMGs may be sensitized to observe a consequence of INPP5D downregulation, just as the microglia in the brain may be sensitized (or primed) by the presence of high levels of Aβ or processes relating to aging. We don’t view this as a weakness but rather a strength of our experimental system, as it allowed us to uncover this biologically relevant relationship between INPP5D and inflammasome activation. Further, iPSC models allow for protein level analyses following pharmacological and genetic manipulations under well-controlled conditions in human cells. Our data suggest that it is imperative to couple RNA profiling with protein profiling in order to get a clear view of the effectors of signaling pathways. However, it is important to interpret these in vitro results in the context of findings from other systems. Here, integrating our findings with data from human tissue aids in our ability to interpret the results. Through the work that is presented in this study, we link INPP5D activity to immune processes in microglia and identify INPP5D as a regulator of NLRP3 inflammasome activity.

Our data suggest that understanding the interplay of signaling mechanisms between INPP5D activity and inflammasome regulation could be key to unraveling aspects of the early stages of microglial involvement in AD. By examining altered transcriptional and proteomic profiles resulting from decreased INPP5D activity, our study suggests three potentially overlapping avenues for linking INPP5D function to microglial activation and elevated risk for AD: (1) altered inflammasome activity within microglia; (2) immune sensing pathways that would affect microglia recognition and engulfment of Aß; and (3) as-of-yet undefined signaling mechanisms that affect the expression of other proteins linked to LOAD pathogenesis through GWAS.

We observed changes of several LOAD GWAS associated proteins in both the 3AC-treated and INPP5D HET models including CD33 and TMEM106B, while several other GWAS associated genes implicated in microglial biology were unchanged. Both CD33 and TREM2 have been linked to INPP5D biology previously^11,69^. CD33 protein and RNA levels were reduced with acute inhibition of INPP5D activity, while *TREM2* RNA levels were not affected (TREM2 was not detected in all samples at the protein level via TMT-MS). Altered levels of other LOAD GWAS related genes following decreased activity of INPP5D indicates that a number of these proteins likely interact functionally with one another.

These results intersect with other aspects of altered microglial function in AD, such as the changes of the microglial subsets specialized in antigen presentation^53^, as our INPP5D heterozygous iMGs display a reduction in the expression of proteins involved in antigen presentation. Microglia recognition and engulfment of Aß peptides are implicated as important processes in AD. Scavenger receptors are putative Aß receptors, important for recognition and uptake of Aß from the extracellular space by microglia. Proteomic and transcriptomic analysis of reduction of INPP5D activity acutely and chronically each revealed significant changes in the expression of scavenger receptors. With acute inhibition, protein levels of MRC1, SCARA1/MSR1, COLEC12, and CLEC7A were reduced. MSR1 is reported as an Aß receptor and decreased MSR1 expression by microglia leads to increased Aß accumulation^70^. This downregulation of scavenger receptors would lead to altered Aß clearance and an accumulation of Aß proteins. However, chronic reduction of INPP5D levels resulted in an *elevation* in protein levels of the scavenger receptors MRC1, COLEC12, CD36 and CD163. CD36 binds to and enhances Aß clearance^71^ and COLEC12 is upregulated in glia near Aß plaques in AD brain and in fAD transgenic mouse models^72^. Further, we observed a reduction in autophagic flux with loss of one copy of INPP5D (Fig. 6h), which could affect Aß degradation following uptake. Other than INPP5D itself, MRC1 was the protein most strongly affected by loss of one copy of INPP5D (Fig. 6a). In previous published studies, MRC1 (CD206) is upregulated in microglia following injury in vivo and following treatment with IL-4 in vitro^73,74^. Along with COLEC12, MRC1 also has been shown to be enriched in border macrophages. A shift in microglial state with chronic reduction in INPP5D is consistent with the depletion of INPP5D observed in cluster 13 (a cluster defined in part by MRC1 and COLEC12 expression) in our analysis of human brain snRNAseq (Fig.1f–i). Thus, modulation of INPP5D activity impacts the profile of scavenger receptors present on the microglia, which in turn may affect both Aß clearance and phagocytosis of other extracellular factors.

Inflammasome activity has been linked to several diseases including AD, thus understanding its method of regulation is critical towards unraveling its role in a number of biological processes^75^. The mechanism of inflammasome regulation and activation appears to be complex and multifaceted, with several aspects remaining to be uncovered. Multiple therapeutic efforts are ongoing to develop and test pharmacologic inhibitors of the NLRP3 inflammasome (such as MCC950, used herein). Previous studies have shown that Aß can activate the inflammasome to increase IL-1ß secretion^44,76^, and that activation of the NLRP3 inflammasome affects plaque levels and contributes to spatial memory defects in APP/PS1 mice^4^. Recent studies linked the NLRP3 inflammasome to the development of tau pathology through phosphatase regulation by the NLRP3 inflammasome and through the worsening of tau pathology through IL-1ß signaling^5^. Here, we show activation of the inflammasome in the absence of external AD-related insults such as neurotoxic Aß or tau. Thus, the activation of the NLRP3 inflammasome following decreased INPP5D activity in microglia may contribute to risk for AD by altering the vulnerability of microglia to additional insults encountered in the brain. Additional studies in the coming years will be necessary to test this hypothesis.

Examining the differences between the vehicle versus 3AC treatment and INPP5D WT versus HET comparisons provide a window into the mechanisms at play following an acute versus chronic decrease in INPP5D activity. With acute reduction of INPP5D activity, the inflammasome is activated with a strong induction of IL-1ß and IL-18 release (up to a 1000-fold increase with 2.5 μM 3AC, Fig. 4c). Extracellular levels of IL-1ß and IL-18 also are elevated with chronic reduction of INPP5D, although the effect is blunted in comparison to acute efficient inhibition (less than 10-fold increase, Fig. 6i, j). This increase is rescued with NLRP3 inhibition (Fig. 6k, l) demonstrating that a low level of inflammasome activation is contributing to this phenotype. With chronic loss of INPP5D, we also observe changes that suggest a negative feedback mechanism as the cells respond to inflammasome activation. This may be mediated in part by the observed upregulation of IL1RN protein, a negative regulator of IL-1R activity. Proteins shown to be rapidly reduced following acute INPP5D inhibition but then elevated with chronic INPP5D loss include MRC1, TMEM106B, PYGL, LAMP1, HK3, H2AFY2, FUCA1, FCGR3B, DMXL2, and BDH2 (Supplementary Fig. 9). Future studies are warranted to determine if the proposed negative feedback mechanism exists and what factors comprise this mechanism.

Data presented herein provide clues to uncovering the signaling cascades that link INPP5D activity to inflammasome regulation. Through the integration of our findings in human microglia with other experimental systems reported in the literature, two candidate pathways emerge. One pathway implicated is through CLEC7A/Dectin-1 signaling and the second through PLA2G7. INPP5D is reported to affect C-Type Lectin Domain Containing 7A (CLEC7A) signaling through FcγR interactions in THP-1 and bone marrow cells^12,13^. In dendritic cells, CLEC7A is involved in a non-canonical inflammasome pathway to induce IL-1ß signaling^77^. Recent studies report that these CLEC7A/inflammasome pathways may be synergistic with the NLRP3 inflammasome^78^. Here, we observed a decrease in CLEC7A protein following INPP5D inhibition coupled to inflammasome activation, suggesting a linkage also exists between these pathways in human microglia (Fig. 3). Secondly, Lipoprotein-associated Phospholipase A2 (PLA2G7) protein levels were rapidly elevated with INPP5D inhibition, and these elevated protein levels also were observed with chronic INPP5D reduction in HET microglia. This elevation was identified through unbiased protein profiling and was validated via western blotting (Fig. 6d, e). PLA2G7 functions by hydrolyzing glycerophospholipids to generate lysophosphatidylcholine (LysoPC), which has proinflammatory effects^79^. PLA2G7 has been primarily studied in its role in atherosclerosis, and an inhibitor of PLA2G7 activity, darapladib, has been proposed as a therapeutic target for atherosclerosis^80–82^. Intriguingly, a recent study showed that darapladib-mediated inhibition of PLA2G7 provides benefit through a downregulation of NLRP3 inflammasome activation in macrophages^83^. Our data show that decreased INPP5D activity elevates protein levels of PLA2G7 which in turn may induce inflammasome activation in human microglia as well. Future experimental efforts will involve utilizing darapladib to investigate whether PLA2G7 inhibition rescues INPP5D inhibition-induced inflammasome activation. For each of these possibilities the lysosome and regulation of autophagy may be playing an important role in inducing inflammasome activation downstream of a reduction of INPP5D function.

Understanding the role of AD-associated microglia genes in disease is a challenging but important aim. In this study, we take a deep dive into the biology of INPP5D in human microglia. Through unbiased analyses of RNA and protein profiles in INPP5D-disrupted microglia, we find that reduction in INPP5D activity is associated with profiles consistent with inflammasome activation. We then validate these findings through a series of targeted pharmacological experiments to show that reduction in INPP5D activity induces the formation of the NLRP3 inflammasome, cleavage of CASP1, and secretion of IL-1ß and IL-18. We also provide an in-depth analysis of human brain tissue across hundreds of individuals. Using a multi-omic approach, we provide rich sets of data that, taken together, suggest that a reduction in function of INPP5D in microglia results in inflammasome activation in the AD brain and that reduced INPP5D activity can have transcriptional consequences on neurons. These datasets provide important clues to unraveling the signaling mechanisms that connect INPP5D to the inflammasome. As NLRP3 inflammasome activity has been linked to many diseases and disorders, the identification of INPP5D as a member of this pathway would have therapeutic significance in other disease fields beyond Alzheimer’s disease.

## Methods

### Human subjects approvals

All work was performed following IRB review and approval through Partners/BWH IRB (2016P000867). Human brain material was obtained from (1) the neuropathology core facility at Massachusetts General Hospital, (2) Rush University Medical Center, (3) Albany Medical Center, and (4) New York Brain Bank. ROS and MAP studies were approved by an Institutional Review Board of Rush University Medical Center. All participants signed an informed consent, an Anatomical Gift Act, and a repository consent to allow their data and biospecimens to be repurposed, and for data acquired from these samples to be published. The ROSMAP studies were approved by an institutional review board of Rush University Medical Center. Each participant signed an informed consent, an Anatomical Gift Act for organ donation, and a separate repository consent allowing for sharing and repurposing of data and biospecimens. IPSC lines were utilized following IRB review and approval through MGB/BWH IRB (#2015P001676).

### Immunostaining of human brain tissue (for INPP5D expression)

Tissue was dissected from the temporal cortex of post-mortem human brain tissue and drop-fixed in 10% formalin for 24 h then buffer exchanged into 30% sucrose for cryopreservation. Tissue was embedded into Tissue-Tek OCT Compound (Sakura Finetek) and frozen for cryosectioning. Tissue was sectioned into 25 μm sections using a cryostat. Antigen retrieval was performed in citrate buffer prior to blocking with 3% BSA and 0.1% triton in PBS. Primary antibodies were prepared in 1% BSA in PBS (INPP5D, 1:100 dilution, Cell Signaling Technologies, 2727S; IBA1, 1:500 dilution, abcam, ab5076) and sections were incubated overnight in this solution at 4 °C. Washes were performed with PBS and incubated in secondaries (Cy2 Donkey anti-Rabbit or Cy3 Donkey anti-Goat, 1:2000 dilution, Jackson Immunoresearch) diluted in PBS for 1 h at room temperature. After washing, sections were incubated in Sudan black in 70% ethanol for 10 min. Sections were washed in PBS and mounted onto glass slides using Vectashield with DAPI for confocal microscopy using a Zeiss LSM710 confocal microscope and acquired using ZEN black software.

### Immunocytochemistry of human brain tissue for quantification

Tissue from the DLPFC (BA9) was obtained from the New York Brain Bank. 6 μm sections of formalin-fixed paraffin-embedded tissue were immunostained for IBA1 (1:100, Wako, 011-27991), INPP5D (1:100, abcam, ab45142) and A*β*42 (1:300, Biolegend, 805501). Alternate brain sections were immunostained for IBA1, Aβ*42*, and ASC (Cell Signaling Technologies, 13833S). Heat-induced epitope retrieval was performed using citrate (pH = 6) in a microwave. The sections were treated with formic acid, blocked, and incubated with primary antibodies. Sections were washed three times with PBS and incubated with fluorochrome conjugated secondary antibodies then with True Black to quench Lipofuscin autofluorescence. Anti-fading reagent with DAPI was used to mount coverslips. For each subject, 30 images of cortical gray matter at magnification 20× (Nikon Eclipse fluorescence microscope) were acquired in a zigzag sequence along the cortical ribbon to ensure that all cortical layers were represented in the quantification in an unbiased manner. The acquired images were analyzed using CellProfiler software^84^. CellProfiler data were imported and aggregated in R. For ASC speck quantification, aggregated data represent 19,916 microglia (identified by IBA1+ staining). Identified microglia were called positive for ASC specks if they were in the upper 6% of ASC expression intensity integrated across the region of interest (defined by IBA1 positivity). Within each brain sample, the percentage of microglia with ASC specks was compared to the average integrated intensity of INPP5D across all identified microglia. To determine whether microglia are associated with plaques, we used the “RelateObjects” module to associate IBA1+ cells with amyloid plaques. IBA1+ cells were considered to be associated with amyloid plaques if they were touching, even partially, the plaques. The morphology of the microglia was measured using the “MeasureObjectSizeShape” and we used the compactness measurement, which is calculated as Perimeter^2^/4*π*Area. A filled circle will have a compactness value of 1, while irregular objects will have a value greater than 1. The more the microglia have a complex morphology (more ramified), the higher the compactness value will be.

All data can be found in Supplementary Data 16.

### Generation of iMGs from iPSCs

iPSCs were maintained on growth-factor reduced Matrigel (Corning) and fed with StemFlex media (ThemoFisher Scientific) and undergo monthly mycoplasma testing to ensure cultures are mycoplasma-free with the LookOut Mycoplasma PCR detection kit (Millipore Sigma). For the differentiation of iPSC-derived microglia-like cells, iPSCs were differentiated using a previously established protocol^22,23^ that was further optimized, as described here. HPCs were generated from iPSCs using the StemDiff Hematopoietic Kit (StemCell Technologies). Cells were replated at day 12 at 100,000 cells per 35 mm well (1 well of a 6 well plate). Cells were plated in 2 mL of iMG media per well: DMEM/ F12, 2× insulin-transferrin-selenite, 1× B27, 0.5× N2, 1× glutamax, 1× non-essential amino acids, 400 μM monothioglycerol, 5 μg/mL insulin. Microglia media is supplemented with fresh cytokines before each use: 100 ng/mL IL-34 (Peprotech), 50 ng/mL TGFβ1 (Millitenyi Biotech), and 25 ng/mL M-CSF (ThermoFisher Scientific). On days 14, 16, 18, 20, and 22, each well was supplemented with 1 mL of iMG media with freshly added cytokines. On day 24, all media is removed from the wells and the cells are dissociated by incubating with 5 min of PBS at room temperature. Both the media and the PBS is centrifuged at 300 × *g* for 5 min to pellet adherent and non-adherent cells. Cells are combined and replated at 100,000 cells per 15.6 mm well (1 well of a 24 well plate) in 1 mL of a 1:1 mixture of old media and fresh iMG media with tri-cytokine cocktail. On days 26, 28, 30, 32, 34, 36, each well is supplemented with 0.5 mL of iMG media with freshly added 3 cytokines. On day 37, all but 0.5 mL of media was removed from each well. Media was centrifuged for 5 min at 300 × *g* to pellet non-adherent cells. Cells are resuspended in iMG media supplemented with 100 ng/mL IL-34, 50 ng/mL TGFβ1, 25 ng/mL M-CSF, 100 ng/mL CD200 (Novoprotein), and 100 ng/mL CX3CL1 (Peprotech) for a total of 1 mL per well. On day 39, cells are fed with microglia media with five-cytokine cocktail (0.5 mL per well). After day 40, cells were ready for experimentation.

### Generation of iA, iN, or iEC samples

The cells were generated in a manner similar to previously published work^26^. NGN2 tetracycline-inducible iPSC neuron lines were generated using a lentiviral transduction protocol as previously described^25^. Plates were coated with poly-ornithine and laminin (10 μg/mL poly-ornithine, 5 μg/mL laminin) 24 h prior to cell plating. Plates were coated with Matrigel basement matrix 2 h prior to cell plating (8.7 µg/cm^2^). Day 4 cells were thawed from stock and plated at 150,000 cells per well with iN media (Neuralbasal media, 1% Glutamax, 0.3% Dextrose, 0.5% MEM NEAA, 2% B27, 10 ng/mL of each BDNF, GDNF, and CNTF, 5 μg/mL puromycin, 2 μg/mL doxycycline, 10 μM Y-27632). Half media changes with the same iN media without Y-27632 were performed every 2-3 days.

Induced astrocytes (iAstros) were generated following a previously published paper^24^ with minor modifications. IPSCs were plated at 95k cells/cm^2^ on a growth factor reduced Matrigel (Corning) coated plate, then were transduced with three lentiviruses – TetO Sox9 puro (Addgene plasmid #117269), TetO Nfib Hygro (Addgene plasmid #117271), and FUdeltaGW-rtTA (Addgene plasmid #19780). The cells were then dissociated with Accutase, plated at 200,000 cells/cm^2^ using Stem- flex and ROCK inhibitor (10 μM) (D0). From D1 to D6, the media was gradually switched from Expansion media (DMEM/F12, 10% FBS, 1% N2 supplement, 1% Glutamax) to FGF media (Neurobasal media, 2% B27, 1% NEAA, 1% Glutamax, 1% FBS, 8 ng/ml FGF, 5 ng/ml CNTF, 10 ng/ml BMP4). On day 8, cells were cryopreserved.

### Immunocytochemistry of cultured cells

iMGs were plated on either glass pre-coated coverslips or 96-well plates at a density of 50,000 cells per coverslip or 16,000 cells per well on day 24 with dissociation as explained above. Cells were fixed with 4% paraformaldehyde and then blocked for 1 h at room temperature with blocking buffer (2% donkey serum, 0.1% triton, in PBS). Primary antibodies were prepared in blocking buffer (INPP5D, 1:100 dilution, Cell Signaling Technologies, 2727S; IBA1, 1:500 dilution, abcam, ab5076; ASC, 1:500 dilution, Cell Signaling Technologies, 13833S; P2RY12, 1:500 dilution, gift from Oleg Butovsky’s lab, TUJ1, 1:100, Millipore, MAB1637) and fixed cells were incubated overnight in primary antibody at 4 °C. Following gentle washes, secondaries diluted in PBS (Cy2 Donkey anti-Rabbit or Cy3 Donkey anti-Goat, 1:2000 dilution, Jackson Immunoresearch) and added for 1 h at room temperature. For cells plated in wells, DAPI was added for a 10-min incubation before final storage in PBS. Coverslips were mounted using Vectashield mounting medium with DAPI. Images were taken using a Zeiss LSM710 confocal microscope and acquired using ZEN black software. Blinding was applied to the examination of ASC spec formation in iMGs with positive controls, negative controls, and 3AC treatment.

### Immunoblotting

iMG, iPSC, iA, iN, or iEC samples were harvested with NP40 buffer (1% NP40, 0.15 mM NaCl, 5 mM Tris, pH 7.4, 1 mM EDTA) supplemented with cOmplete protease inhibitor cocktail (Roche) and PhoSTOP phosphatase inhibitors (Roche). Brain extract sample preparation is explained below. Samples were spun down at 15,000 × *g* for 15 min at 4 °C on a tabletop centrifuge and then the supernatant was collected for protein quantification using the Pierce BCA protein assay kit. Samples were prepared using 4× Protein Sample Loading Buffer (Licor) as per manufacturer instructions and then run on NuPAGE 4–12% Bis-Tris mini or midi protein gels with either 1× MOPS or 1× MES buffer. Western blot transfer was performed on ice using Tris Glycine transfer buffer onto nitrocellulose membrane and then blocked for 1 h at room temperature with Intercept Blocking Buffer (Licor). Membranes were incubated with primary antibodies diluted in blocking buffer over night at 4 °C (INPP5D, 1:500 dilution, Cell Signaling Technologies, 2727S; INPP5D 1:1000, abcam, ab45142; IBA1, 1:500 dilution, abcam, ab5076; GAPDH, 1:10,000 dilution, Proteintech, 60004; PAFAH/PLA2G7, 1:500 dilution, Proteintech, 15526-1-AP; MRC1, 1:1000 dilution, abcam, ab64693; COLEC12, 1:500 dilution, R&D Systems, AF3130; CASP1, 1:1000 dilution, Cell Signaling Technologies, 3866S; Cleaved CASP1, 1:500 dilution, Adipogen, AG-20B-0048-C100; ASC, 1;1000 dilution, Cell Signaling Technologies, 13833S; NLRP3, 1:1000 dilution, Cell Signaling Technologies, 15101S; GSDMD, 1:1000 dilution, abcam, ab210070; LC3, 1:1000 dilution, MBL International Corporation, M186-3). Washes were performed with TBS buffer with 0.01% Tween-20 (TBS-T). Blots were incubated with secondary antibodies diluted in TBS-T buffer (800CW Donkey anti-Rabbit, 800CW Donkey anti-Goat, 680RD Donkey anti-Mouse, 1:10,000 dilution, Licor Biosciences) for 1 h at room temperature. Washes were performed again in TBS-T before final storage in TBS for imaging using an LICOR Odyssey CLx imager. Bands were quantified using Image Studio Lite and signals normalized to GAPDH or IBA1 levels. For western blot analysis for Urea or TBS brain extracts, experimenters performing the western blots were blind to AD diagnosis of patient samples and ROUT Q = 10% was used to eliminate outliers.

### Dissociation for single nucleus RNA sequencing and library generation

All media were removed from the wells and the cells are dissociated by incubating with 5 min of PBS at room temperature. Both the media and the PBS are centrifuged for 5 min at 300 × *g* and the pellets were combined and washed once in ice-cold PBS with 0.04% BSA. The suspension was centrifuged for 5 min at 500 × *g* to pellet cells that were then lysed on ice for 15 min with lysis buffer: 10 mM Tris, 0.49% CHAPS, 0.1% BSA, 21 mM magnesium chloride, 1 mM calcium chloride, 146 mM sodium chloride. The nuclei were pelleted through centrifugation 5 min at 500 × *g* and resuspended in PBS + 1% BSA for counting prior to loading into the 10X following manufacturer’s instructions.

Single nucleus suspensions at 1000 cells/μL were used to generate gel emulsion bead suspensions using the 10xGenomics Chromium controller and NextGEM 3′ reagents v3.1 (10X Genomics, San Francisco, CA) with a targeted recovery of 10,000 cells. Single cell libraries were generated following the “Chromium NextGEM” protocol (CG000204 Rev D). A TapeStation 4200 was used to assay library quantity and size distribution. Libraries were sequenced at the New York Genome Center using a NovaSeq at a depth of 4 × 10^8^ reads per sample (4 × 10^4^ reads per cell). Fastq files were processed using the 10xGenomics CellRanger pipeline and standard CellRanger outputs were visualized using the Loupe Browser.

### Single nucleus RNAseq analysis of human brain and iMGs

The 10x Genomics CellRanger pipeline “filtered_feature_bc_matrix” output from two iPSC-derived microglia sample runs (batches) were merged for downstream single nucleus RNA-seq data analysis using the Seurat v4.0.3 package for R. The data object was filtered by keeping only genes detected in at least 10 nuclei, and nuclei were filtered to only include those with detection of at least 100 genes. The percentages of mitochondrial genes and ribosomal protein-related genes were calculated, after which mitochondrial related genes and sex chromosome related genes (XIST, UTY) were removed. The data object was further filtered, keeping only nuclei with less than 5% mitochondrial related genes, with more than 1000 and less than 25,000 unique mapped transcripts (Unique Molecular Identifiers – UMIs).

To assess cell type similarity of microglia, post-mortem human nuclei from individuals without Alzheimer’s Disease diagnoses were used, from the Cain et al. publication^29^. Glutamatergic neurons, astrocytes and microglia from this post-mortem dataset were reprocessed by removing mitochondrial related genes and sex chromosome related genes (XIST, UTY), and these nuclei were then merged with the iMG data object.

For normalization and integration of this merged data object, the Seurat function “SCTransform” was used to identify 3000 variable genes in all nuclei and regress out UMI counts. 25 principal components were used to resolve features in the merged data object and the Harmony function “RunHarmony” was used to integrate nuclei anchored by cell types. 25 Harmony-derived dimensions and 30 k-nearest neighbors were used to embed nuclei into a Uniform Manifold Approximation and Projection (UMAP) space for visualization. In parallel, these Harmony dimensions were used to identify cell clusters using the standard Louvain community detection algorithm implemented in Seurat. Finally, the Seurat function “FindAllMarkers” was used to find gene markers for cell clusters. This function calculates significantly up- or downregulated genes in each cluster versus all the others, and reports the raw Wilcoxon rank sum test *p*-value, and the FDR-adjusted value.

### Brain extract preparation

Tris Buffered Saline (TBS: 20 mM Tris-HCl, 150 mM NaCl, pH 7.4) brain extracts were prepared using the method previously described^26^. Briefly, the prefrontal cortex (PFC) tissue was dissected to isolate the gray matter, which was homogenized in ice-cold TBS at a ratio of 1:4 tissue weight to buffer volume within a Dounce homogenizer. This suspension was then subject to ultra-centrifugation at 1.75 × 10^5^ × *g* in a TL100 centrifuge (Beckman Coulter) to pellet cellular debris. Urea brain extracts were prepared in 8 M urea and prepared as per a previous study^34^. Experimenter was blinded to diagnosis for Western blotting quantification, wherein wells were excluded that showed “pinched” bands or abnormally high background. Those samples were re-run and quantified. Following quantification, levels of INPP5D and GAPDH were normalized to internal controls across blots.

### iMG treatments

3AC (Echelon Biosciences) was reconstituted in 100% ethanol as per manufacturer instructions. 3AC (1.25 μM, 2.5 μM, or 5 μM) treatment was performed for 6 h. LPS, Nigericin, VX-765 (Invivogen), Ac-YVAD-cmk (Invivogen), and MCC950 (Invivogen) were reconstituted as per manufacturer instructions. iMGs were treated with LPS (100 ng/mL) for 6 h and VX-765 (25 μM) and MCC950 (10 μM) were treated with 3AC (5 μM) for 6 h prior to harvesting. For Ac-YVAD-cmk treatments, iMGs were pre-treated first for an hour with Ac-YVAD-cmk (20, 40, 80 μM) prior to 3AC (5 μM) treatment for 6 h and harvesting. For ASC immunostaining, cells were treated with LPS (100 ng/mL) for 3 h and then Nigericin (10 μM) for 1 h prior to fixation with 4% paraformaldehyde. Conditioned media was collected from iMGs following treatment to measure for levels of secreted IL-1ß and IL-18. To measure cytotoxicity, LDH-Glo Cytotoxicity Assay was used as per the manufacturer’s instructions (Promega, J2380). For INPP5D overexpression, iMGs were transduced with either control or INPP5D OE (Origene, RC212176L1V; MOI = 5) co-delivered with Vpx VLPs as described in ref. ^61^. The cells were transduced on d42 of differentiation and harvested following 72 h following transduction on d45 for analyses.

### ELISAs for secreted cytokine and APOE measurements

Conditioned media was assayed for cytokine levels using the Meso Scale Discovery V-PLEX proinflammatory panel (Meso Scale Discovery, K15049D-1)or the U-PLEX dual IL-1ß and IL-18 assay (Meso Sale Discovery, K15067L-2). For INPP5D WT and HET conditioned media, the media was concentrated at room temp using a Savant SpeedVac SPD1030 Vacuum Concentrator for 2 h to concentrate the media tenfold prior to loading onto the dual IL-1ß and IL-18 assay. For Urea brain extracts, samples were diluted 1:8 in the sample buffer provided by the MSD U-PLEX dual IL-1ß and IL-18 panel. One sample was run on all plates to normalize across plates. For measuring levels of secreted APOE, 48 h conditioned media was collected prior to harvest for either iMG or iN and iMG co-cultures. Extracellular APOE was measured using MSD R-PLEX Human ApoE Assay (Meso Scale Discovery, K1512IR-2).

### Bulk RNA sequencing

For INPP5D acute inhibition, samples include iMGs from 2 genetic backgrounds (BR01, BR33), treated with 3AC (1.25 μM 3AC, 6 h) or vehicle (ethanol). INPP5D HETs were in the BR33 background. The INPP5D biallelic loss-of-function monoclones were in the BR24 background and the INPP5D overexpression samples were also collected from the BR24 background. For all samples 500 ngs of total RNA input was sequenced through the Genewiz polyA selection, HiSeq 2 × 150 single index sequencing platform. RNAseq reads were quality tested using fastqc, quality trimmed then quantified using the Kallisto pseudoalignment quantification program (v0.43.1)^85^ running 50 bootstraps against a Kallisto index generated from GRCh38. Kallisto quantified samples were analyzed using the “Sleuth” package (v0.30.0) in R Studio (v3.6.1 of R; v1.2.5019 of R Studio)^86^. Expression values were exported from the Sleuth object as normalized TPM values. The final RNAseq master expression matrix has 16 samples and quantifies expression of 43,122 genes. To identify differentially expressed genes the above matrix was filtered to remove low expressers (greater than 5 TPM in at least 2 samples), leaving 13,532 quantified genes. Conditions were compared by linear modeling with empirical Bayesian analysis using the “limma” package^87^ (v3.54.2).

### TMT proteomics and data analysis

#### Sample processing

Media were removed from the wells and iMGs were dissociated by incubating with 5 min of PBS at room temperature. Cells were pelleted with a 500 × *g*, 5 min spin at room temperature, and flash frozen in liquid nitrogen for transport. Each cell pellet was individually homogenized in 300 μL of urea lysis buffer (8 M urea, 100 mM NaHPO4, pH 8.5), including 5 μL (100x stock) HALT protease and phosphatase inhibitor cocktail (Pierce). All homogenization was performed using a Bullet Blender (Next Advance) according to manufacturer protocols. Briefly, each tissue piece was added to Urea lysis buffer in a 1.5 mL Rino tube (Next Advance) harboring 750 mg stainless steel beads (0.9–2 mm in diameter) and blended twice for 5 min intervals in the cold room (4 °C). Protein supernatants were transferred to 1.5 mL Eppendorf tubes and sonicated (Sonic Dismembrator, Fisher Scientific) 3 times for 5 s with 15 s intervals of rest at 30% amplitude to disrupt nucleic acids and subsequently vortexed. Protein concentration was determined by the bicinchoninic acid (BCA) method, and samples were frozen in aliquots at −80 °C. Protein homogenates (50 μg) treated with 1 mM dithiothreitol (DTT) at 25 °C for 30 min, followed by 5 mM iodoacetamide (IAA) at 25 °C for 30 min in the dark. Protein mixture was digested overnight with 1:100 (w/w) lysyl endopeptidase (Wako) at room temperature. The samples were then diluted with 50 mM NH4HCO3 to a final concentration of less than 2 M urea and then and further digested overnight with 1:50 (w/w) trypsin (Promega) at 25 °C. Resulting peptides were desalted with a Sep-Pak C18 column (Waters) and dried under vacuum.

#### Tandem mass tag (TMT) labeling

Peptides were reconstituted in 100 μl of 100 mM triethyl ammonium bicarbonate (TEAB) and labeling performed as previously described^88,89^ using TMTPro isobaric tags (Thermofisher Scientific, A44520). Briefly, the TMT labeling reagents were equilibrated to room temperature, and anhydrous ACN (200 μL) was added to each reagent channel. Each channel was gently vortexed for 5 min, and then 20 μL from each TMT channel was transferred to the peptide solutions and allowed to incubate for 1 h at room temperature. The reaction was quenched with 5% (vol/vol) hydroxylamine (5 μl) (Pierce). All 16 channels were then combined and dried by SpeedVac (LabConco) to approximately 100 μL and diluted with 1 mL of 0.1% (vol/vol) TFA, then acidified to a final concentration of 1% (vol/vol) FA and 0.1% (vol/vol) TFA. Peptides were desalted with a 60 mg HLB plate (Waters). The eluates were then dried to completeness.

#### High pH fractionation

High pH fractionation was performed essentially as described^90^ with slight modification. Dried samples were re-suspended in high pH loading buffer (0.07% vol/vol NH4OH, 0.045% vol/vol FA, 2% vol/vol ACN) and loaded onto a Water’s BEH (2.1 mm × 150 mm with 1.7 µm beads). A Thermo Vanquish UPLC system was used to carry out the fractionation. Solvent A consisted of 0.0175% (vol/vol) NH_4_OH, 0.01125% (vol/vol) FA, and 2% (vol/vol) ACN; solvent B consisted of 0.0175% (vol/vol) NH_4_OH, 0.01125% (vol/vol) FA, and 90% (vol/vol) ACN. The sample elution was performed over a 25 min gradient with a flow rate of 0.6 mL/min with a gradient from 0 to 50% B. A total of 96 individual equal volume fractions were collected across the gradient and dried to completeness using a vacuum centrifugation.

#### Liquid chromatography tandem mass spectrometry

All samples were analyzed on the Evosep One system using an in-house packed 15 cm, 75 μm i.d. capillary column with 1.9 μm Reprosil-Pur C18 beads (Dr. Maisch, Ammerbuch, Germany) using the pre-programmed 21 min gradient (60 samples per day) essentially as described^91^. Mass spectrometry was performed with a high-field asymmetric waveform ion mobility spectrometry (FAIMS) Pro equipped Orbitrap Eclipse (Thermo) in positive ion mode using data-dependent acquisition with 2 s top speed cycles. Each cycle consisted of one full MS scan followed by as many MS/MS events that could fit within the given 2 second cycle time limit. MS scans were collected at a resolution of 120,000 (410–1600 m/z range, 4 × 10^5^ AGC, 50 ms maximum ion injection time, FAIMS compensation voltage of −45). All higher energy collision-induced dissociation (HCD) MS/MS spectra were acquired at a resolution of 30,000 (0.7 m/z isolation width, 35% collision energy, 1.25 × 10^5^ AGC target, 54 ms maximum ion time, TurboTMT on). Dynamic exclusion was set to exclude previously sequenced peaks for 20 s within a 10-ppm isolation window.

#### Data processing protocol

All raw files were searched using Thermo’s Proteome Discoverer suite (version 2.4.1.15) with Sequest HT. The spectra were searched against a human Uniprot database downloaded August 2020 (86395 target sequences). Search parameters included 10 ppm precursor mass window, 0.05 Da product mass window, dynamic modifications methionine (+15.995 Da), deamidated asparagine and glutamine (+0.984 Da), phosphorylated serine, threonine, and tyrosine (+79.966 Da), and static modifications for carbamidomethyl cysteines (+57.021 Da) and N-terminal and Lysine-tagged TMT (+304.207 Da). Percolator was used to filter PSMs to 0.1%. Peptides were grouped using strict parsimony and only razor and unique peptides were used for protein level quantitation. Reporter ions were quantified from MS2 scans using an integration tolerance of 20 ppm with the most confident centroid setting. Only unique and razor (i.e., parsimonious) peptides were considered for quantification.

Proteomic data were filtered to remove any proteins with missing. The ComBat algorithm in the SVA (v3.34.0) package in R was used to remove variance induced by differentiation round. Differentially Expressed Proteins were identified by linear modeling with empirical Bayesian analysis using the “DEP” package^92^ in R.

### Real-time quantitative PCR

RNA was harvested from iMGs and purified using kit instructions (PureLink RNA Mini Kit, Invitrogen). cDNA was generated from RNA using SuperScript II Reverse Transcriptase. Assay was performed using Power SYBR™ Green PCR Master Mix on an Applied Biosciences Vii7a Real-time PCR machine. Samples were assayed with 3 technical replicates and analyzed using the DDC_T_ method and expression was normalized to GAPDH expression. All primer sequences are detailed in Supplementary Data 15.

### CRISPR targeting to generate INPP5D heterozygote and biallelic loss-of-function iPSCs

Guide RNAs were designed using the Broad Institute CRISPick sgRNA design tool^93,94^. The guide RNAs were ligated into plasmid backbone (pXPR_003, Addgene) and the sgRNA plasmid along with a plasmid containing Cas9 (pLX_311-Cas9, Addgene) were transfected into iPSCs using Lipofectamine 3000. Gene editing was confirmed using the GeneArt Genomic Cleavage Detection Kit and the target locus was amplified with PCR and sent for sequencing. Decreased *INPP5D* expression was confirmed using qPCR and western blotting for mRNA and protein levels following successful differentiation to iMGs. All guide RNA and primer sequences are detailed in Supplementary Data 15.

### Autophagic flux measurements

iMGs were treated with either vehicle (DMSO) or 100 nM bafilomycin (Tocris) for either 24 or 6 h. Cells were then harvested using NP40 lysis buffer as described previously and levels of INPP5D, LC3, and GAPDH were determined and quantified via western blotting (LC3, 1:1000, MBL International Corporation, M186-3).

### Co-culturing of iMGs with iNs

Neurons were differentiated from two different genetic backgrounds (BR65, BR99)^26^. Each was co-cultured with either INPP5D-HET or INPP5D WT (CRISPR monoclonal control) from a single genetic background (BR33)^26^. For co-culture, at d40 of differentiation, iMGs were dissociated from culture by incubating cells with room temperature PBS for 5 min. Cells were centrifuged for 5 min at 300 × *g* to pellet the cells before resuspension in iMG media with the five-cytokine cocktail. Counting was performed to ascertain cell density. iMGs were plated onto the ongoing neuronal culture (at d15 of differentiation) in an iN-iMG co-culture media (1:1 Neuralbasal media:iMG base media, 10% B27, 10 ng/mL of each BDNF, GDNF, and CNTF, 100 ng/mL of each IL-34, CX3CL1, and CD200, 50 ng/mL TGFβ1, and 25 ng/mL M-CSF). Cells were then co-cultured for 48 h prior to dissociation for 10x scRNAseq.

### Dissociation for single cell RNA sequencing and library generation and analysis (co-culture)

All media were removed from all four culture conditions (WT iMG+BR65 iNs, HET iMG+BR65 iNs, WT iMG+BR99 iNs, HET iMG+BR99 iNs) and the cells were dissociated with a 1:1 mixture of warm trypsin-EDTA:cold StemPro Accutase Cell Dissociation Reagent and trypsin was quenched with warm neurobasal media (NBM) prior to collection of cell suspension. Cell suspensions were centrifuged for 5 min at 300 × *g* and resuspended in NBM. Cell pellet was triturated gently with a p1000 tip and passed through a 30 μM filter to remove neurites and debris. Cell suspension was centrifuged for 5 min at 300 × *g* and resuspended before resuspended in ice-cold PBS with 0.04% BSA for counting prior to loading into the 10X following manufacturer’s instructions. Each co-culture was loaded onto an individual 10X Chromium Chip well (16,000 cells per well) and resulting emulsions were used to generate scRNAseq libraries using the Chromium Next GEM Single Cell 3′ v3.1 chemistry.

Libraries were multiplexed and sequenced on three lanes of a NovaSeq resulting in 8.3BN total post-normalization reads for 46,046 cells. Fastq files were mapped using the 10xGenomics Cellranger (v7.0.0) pipeline and a GRCh38 index and resulted in >80,000 mapped reads per cell. Mappings and counts were analyzed using the Seurat package (v4.0.4) in R (v4.0.3) using RStudio (v1.4.1103). Briefly, imported Cellranger data were filtered to remove cells with <3000 or > 10000 mapped genes and with > 20% mapping to mitochondrial genes, and to remove suspected doublets. The remaining dataset has 24717 cells (11201 iMGs and 13517 iNs) with an average number of mapped genes detected per cell of 5836 for iMGs and 6596 for iNs, and average total UMIs (unique RNA strands captured) per cell of 37027 (iMGs) and 37112 (iNs). After filtering, normalized and scaled data were clustered using the 3000 most highly variable features. Uniform Manifold Approximation and Projection (UMAP) was run on the first 20 principal components. DEGs between cell groups were identified using FindMarkers function in Seurat using the Wilcoxon Rank Sum test with a multiple comparisons adjusted (FDR) *p*-value cutoff of 0.05.

### Statistics and reproducibility

Statistical analyses of the data varied by data type and were conducted as outlined within each figure legend. All experimental results presented were reproduced across independent experiments as noted within each figure legend. For cell model data, no statistical method was used to predetermine sample size, however, we employed our previous experience using iPSC technology and guidance from the international Society for Stem Cell Research (ISSCR) to include at least three differentiations for each reported result, with multiple replicate wells per differentiation. For human brain tissue data from ROS and MAP, we utilized all samples and data available for the study that met the diagnostic criteria stated. No data were excluded from the analyses unless otherwise noted in specific methods descriptions. The Investigators were blinded to allocation during acquisition of microscopy data.

### Reporting summary

Further information on research design is available in the Nature Portfolio Reporting Summary linked to this article.

### Supplementary information

Supplementary Information

Peer Review File

Description of Additional Supplementary Files

Supplementary Data 1-24

Reporting Summary

### Source data

Source Data

## Data Availability

All RNAseq data generated in this study have been deposited in the NCBI-GEO database under accession code (GSE244209). The proteomic data generated in this study are provided as a Supplementary Dataset, and also can be found on the AMP-AD Knowledge Portal (10.7303/syn52052842) and the PRIDE database (PXD046040). All source data not presented in Supplemental Datasets are provided within the Source Data file. An interactive data viewer for RNAseq and proteomic data presented in this study from iPSC-derived microglia also can be found here: https://youngpearselab.shinyapps.io/inpp5d_imgls/. Additional phenotypic data from ROS and MAP cohorts can be requested at www.radc.rush.edu. Previously published data from brain tissue data (citations found within) can be found on the AMP-AD Knowledge Portal: Genotype data, used here to determine INPP5D SNP profiles: 10.1038/mp.2017.20 Human brain RNAseq used here to examine INPP5D RNA levels in human brain: 10.1038/s41593-018-0154-9 Human brain TMT-MS, used here to examine INPP5D protein levels in human brain: 10.1101/806752. Source data are provided with this paper.
