# Supplementary material for: INPP5D regulates inflammasome activation in human microglia
Source: Nat Commun. 2023 Nov 29;14:7552. doi: 10.1038/s41467-023-42819-w (PMC10684891; doi:10.1038/s41467-023-42819-w)

Supplementary Information

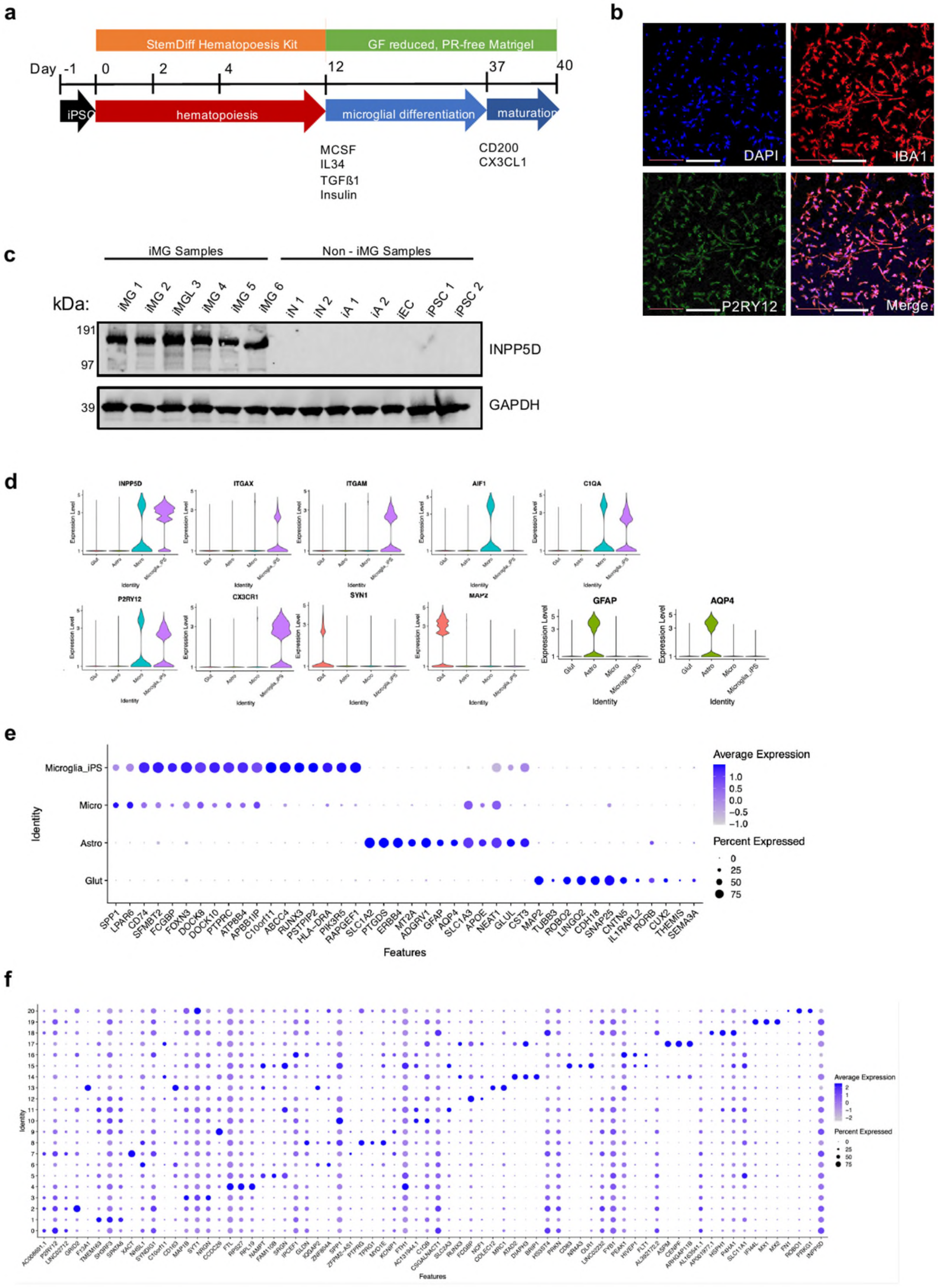

Supplementary Figure 1: Characterization of iPSC-derived microglia. **a.** iMG differentiation timeline[22,23]. **b.** Immunostaining of iMGs for P2RY12 and IBA1. Representative images are shown of over 10 differentiations and immunostaining rounds. Scale bars = 200 µm. **c.** Western blot of INPP5D and GAPDH in iMGs, iPSC-derived neurons (iN), iPSC-derived astrocytes (iA), iPSC-derived endothelial cells, and undifferentiated iPSCs. iMG samples are from 6 differentiations of two different genetic backgrounds. WB is representative of observations of specific INPP5D expression in microglia relative to neurons and astrocytes in over three differentiations. **d.** Violin plots of cell fate marker genes for microglia, neurons, and astrocytes from single nucleus sequencing of iMGs (Microglia_iPS), and glutamatergic neurons (Glut), astrocytes (Astro), and microglia (Micro) from dorsolateral prefrontal cortex (dlpfc) from 12 human postmortem brain samples[34] and iMGs. **e.** Average expression of cell fate markers genes across the iMGs and microglia, astrocytes, and glutamatergic neurons from postmortem brain samples. **f.** Data from microglia subcluster from the snRNAseq in **Fig.1c,d** were isolated and re-clustered to examine microglial subsets. Shown is a bubble plot of top genes that define each cluster in **Fig. 1f-h.**

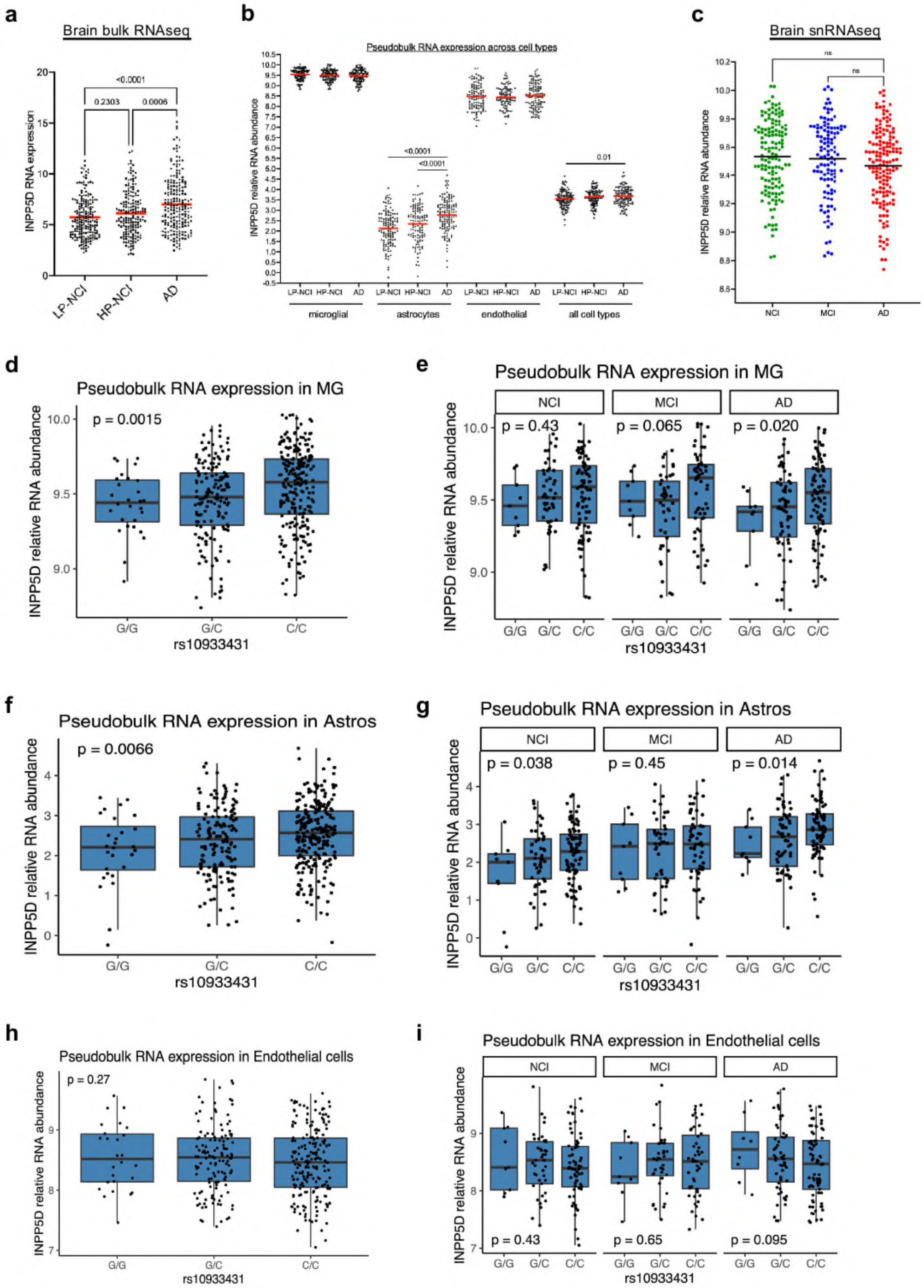

Supplementary Figure 2: Bulk and pseudobulk RNAseq analysis of human brain tissue of INPP5D expression across cell types. **a.** Relative levels of INPP5D RNA as determined by RNA sequencing of 584 ROSMAP human PFC samples (198 LP-NCI, 178 HPNCI, 208 AD; source data from[33]). One-way ANOVA with Tukey's multiple comparisons test. **b-i.** A recent study has performed snRNAseq on a larger cohort of ROS and MAP brain tissue, with brain tissue from over 424 participants analyzed[37,38]. In this analysis[38], pseudobulk levels were calculated for each cell type across individuals and INPP5D quantification is plotted here by diagnosis (**b,c**) and by variant calls for rs10933431, the lead SNP at the INPP5D locus for LOAD association (**d-i**). Statistics performed within a cell type, one-way ANOVA with Dunnett's multiple comparisons test. **d-i.** Examination of associations between rs10933431 variants and INPP5D RNA levels within cell types. Boxplots are shown of INPP5D RNA expression within each cell type. Frequencies of genotype of rs10933431 are G/G (n = 28), G/C (n = 158), C/C (n = 238). Allele count was significantly associated with INPP5D expression by a linear model adjusted for sex and age in microglia as shown.

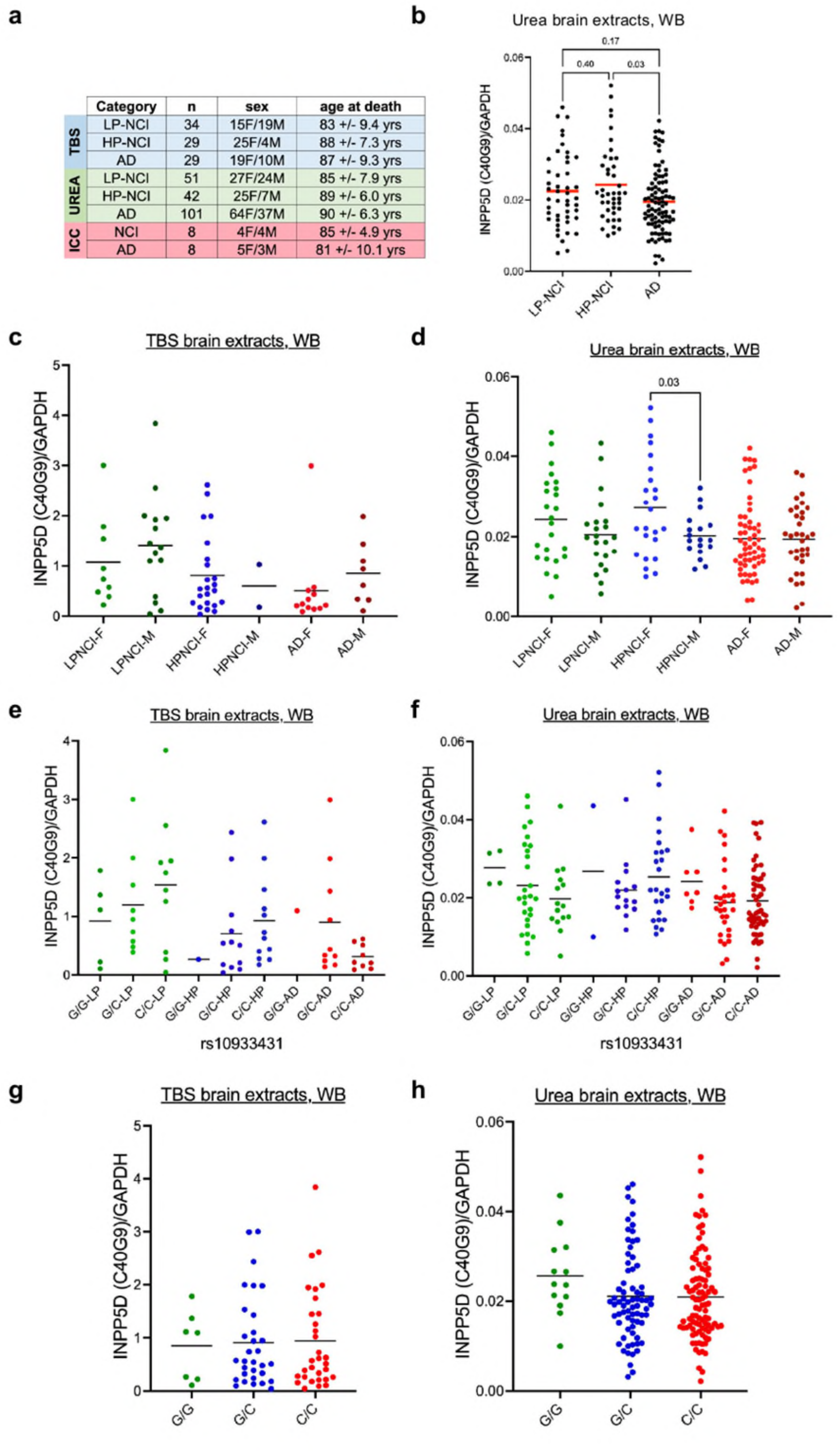

	Category	n	sex	age at death
TBS	LP-NCI	34	15F/19M	83 +/- 9.4 yrs
TBS	HP-NCI	29	25F/4M	88 +/- 7.3 yrs
TBS	AD	29	19F/10M	87 +/- 9.3 yrs
UREA	LP-NCI	51	27F/24M	85 +/- 7.9 yrs
UREA	HP-NCI	42	25F/7M	89 +/- 6.0 yrs
UREA	AD	101	64F/37M	90 +/- 6.3 yrs
ICC	NCI	8	4F/4M	85 +/- 4.9 yrs
ICC	AD	8	5F/3M	81 +/- 10.1 yrs

Supplementary Figure 3: Analyses of INPP5D protein levels by sex, diagnostic category, and genotype in human brain tissue. **a.** A summary table detailing the brain samples assayed in Fig. **2i-o. b**. Western blot quantification of INPP5D and GAPDH from 181 ROSMAP human brain samples extracted in urea. One-way ANOVA with Holm-Sídák's multiple comparisons test multiple comparisons test. **c-h**. Data from Fig.2j and panel **b** is replotted by sex and diagnostic category and by genotype. *p < 0.05.

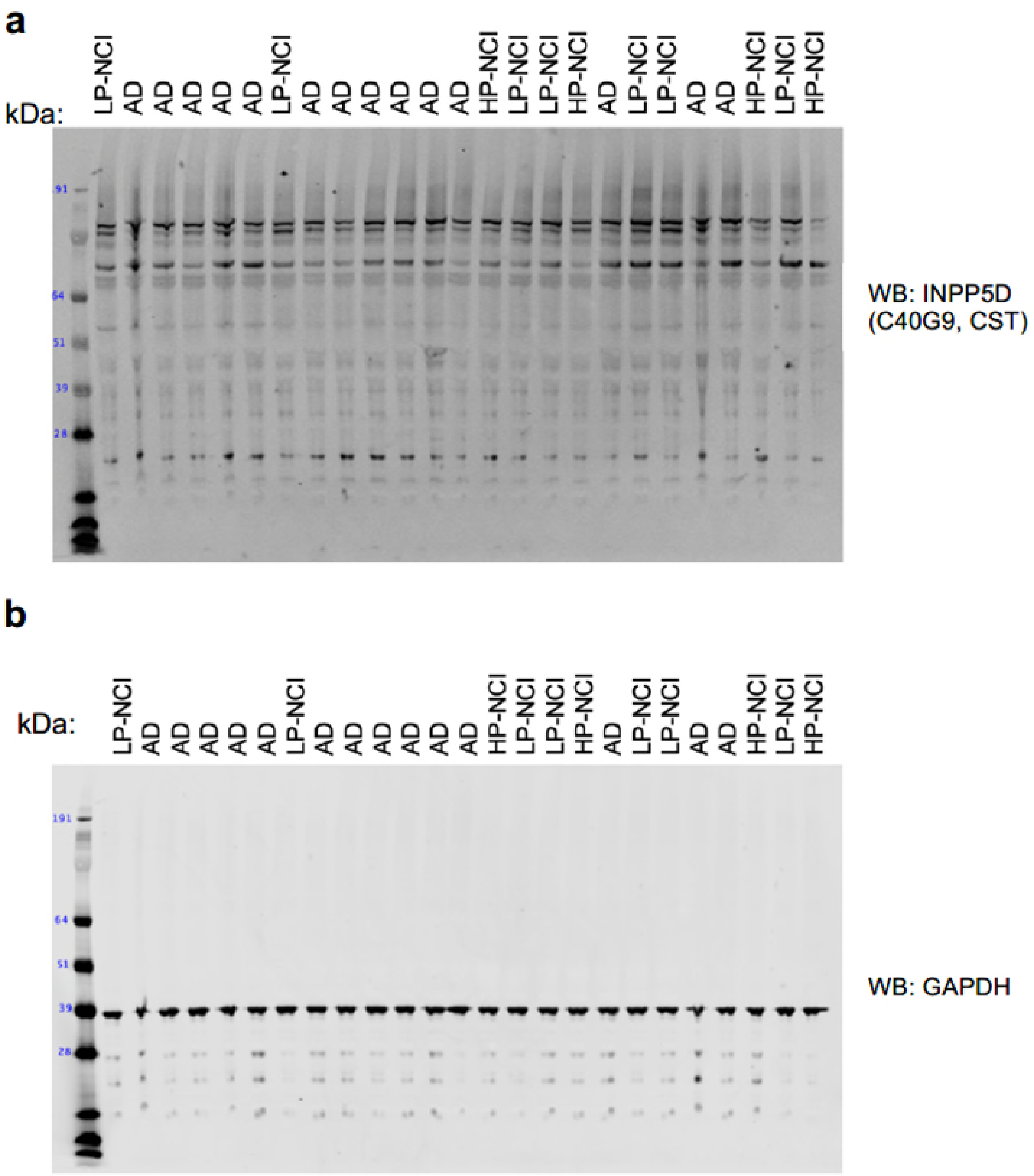

Supplementary Figure 4: Urea brain extract representative western blot. Representative western blots of urea extracts from postmortem human brain for INPP5D and GAPDH levels showing the banding patterns observed. Observation made repeatedly, see Supplementary Data 13 for quantifications for each brain analyzed.

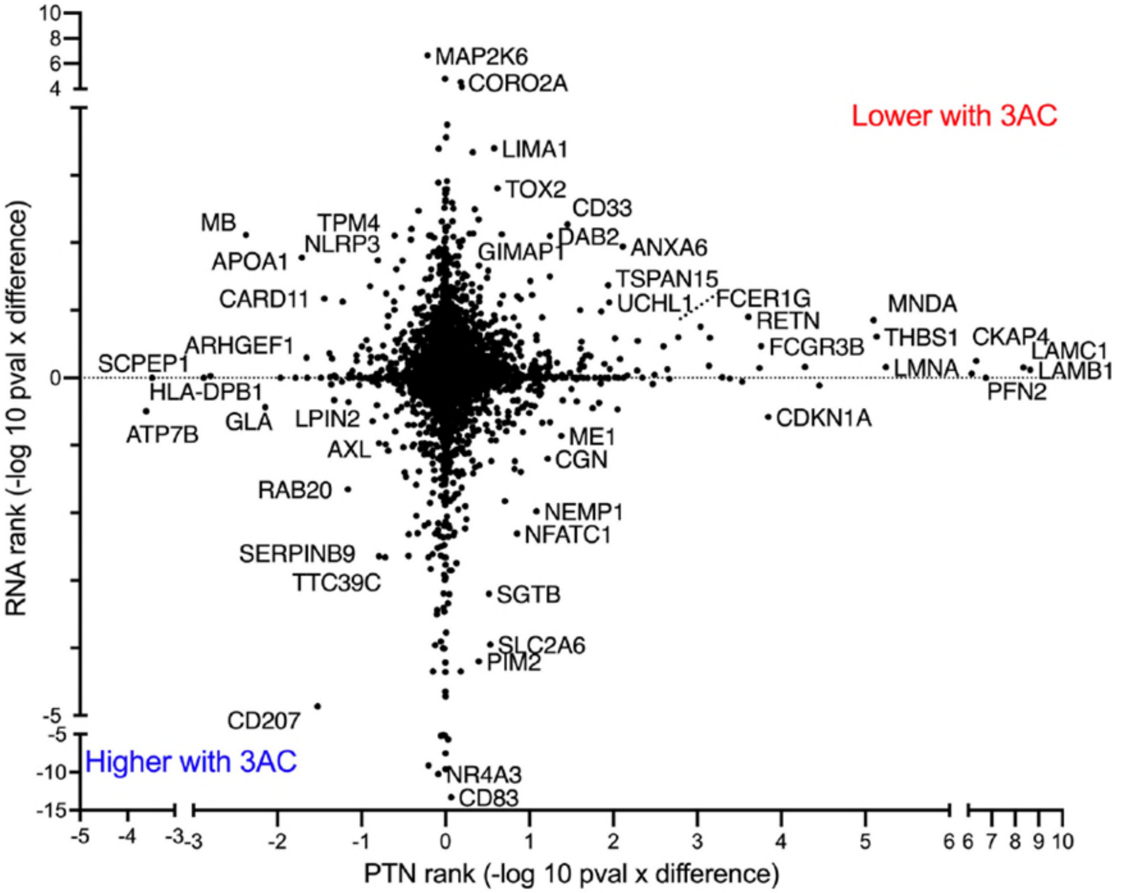

Supplementary Figure 5: Integration of effects of 3AC in iMGs on RNA and protein levels. Data from **Fig.3a and 3b** were combined to show concordant and discordant effects on RNA and protein levels by gene. Comparison of DEG (RNAseq) and DEP (TMT-MS) analyses highlights genes concordantly and discordantly regulated with 3AC treatment of iMGs for 6hrs. Following 3AC versus vehicle comparisons (**Fig. 3a,b**), each gene was ranked by taking the - log10 of the p-value multiplied by the difference in expression within 3AC and vehicle treated samples. The rank in RNA versus protein-level analyses are graphed. Complete data sets found in Supplementary Data 17 and 19.

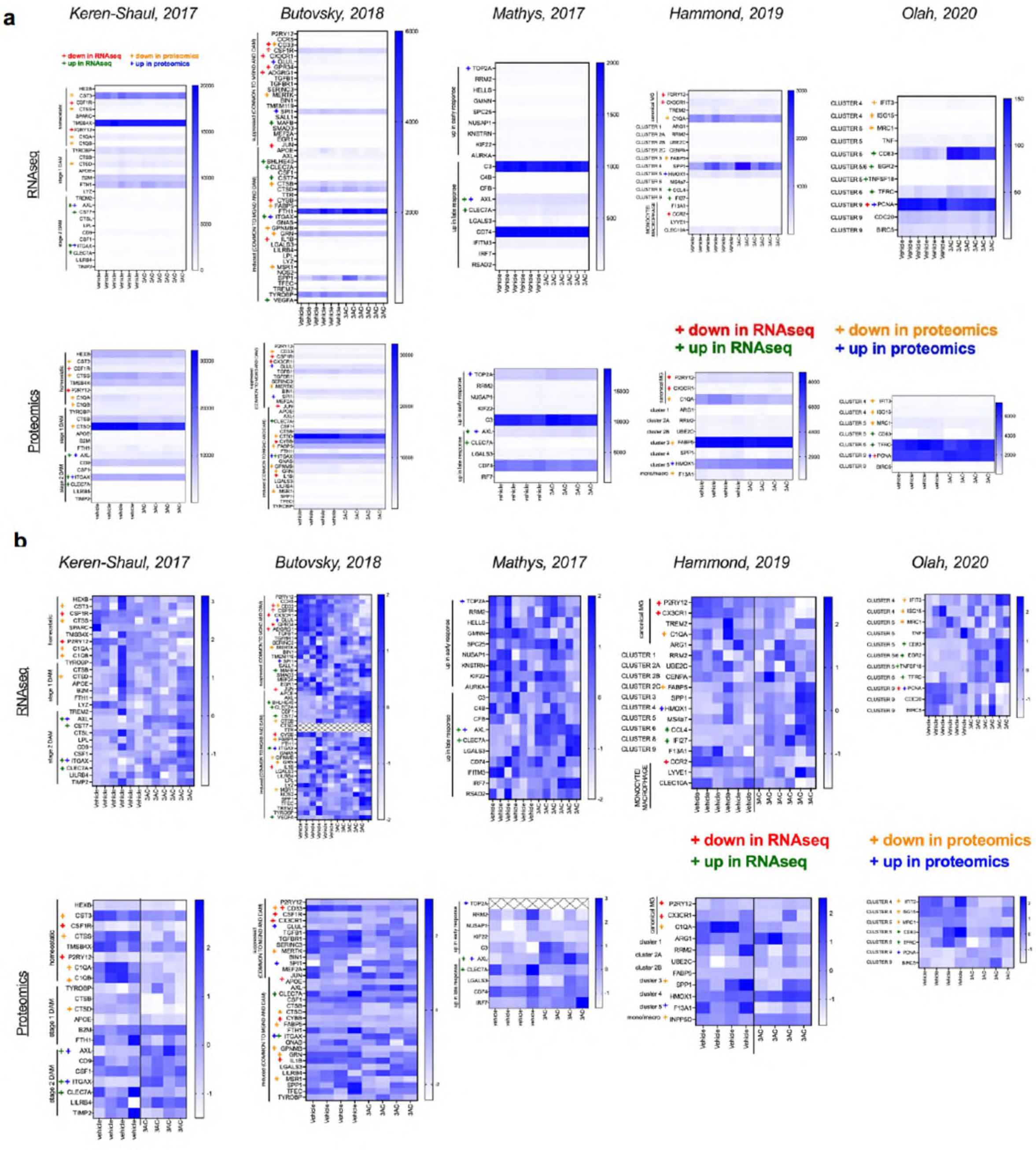

Supplementary Figure 6: Heat map of genes defining microglial states in different published studies. Relative expression of specific genes from **Fig. 3a and 3b** are shown in heat maps showing either relative abundance (TPM, **a**) or else by z-scores (**b**) for ease of viewing differences. Complete data sets found in Supplementary Data 17 and 19.

a

Cell Line	Sex	Diagnosis	APOE	rs10933431	rs35349669	IL-1B (fold change)	IL-18 (fold change)
BR01	female	LP-NCI	2,3	1	0	3.25	6.68
BR09	male	LP-NCI	3,3	0	0	69.44	412.92
BR21	female	LP-NCI	3,3	1	0	31.18	321.97
BR33	male	LP-NCI	3,3	0	0	4.41	3.09
BR68	male	LP-NCI	3,3	2	0	71.01	43.34
BR93	male	LP-NCI	3,3	2	1	3.53	1.04
BR95	female	LP-NCI	2,3	2	1	23.43	22.06
BR98	male	AD	3,3	1	0	13.46	37.07
BR103	female	AD	3,3	2	2	8.18	12.11

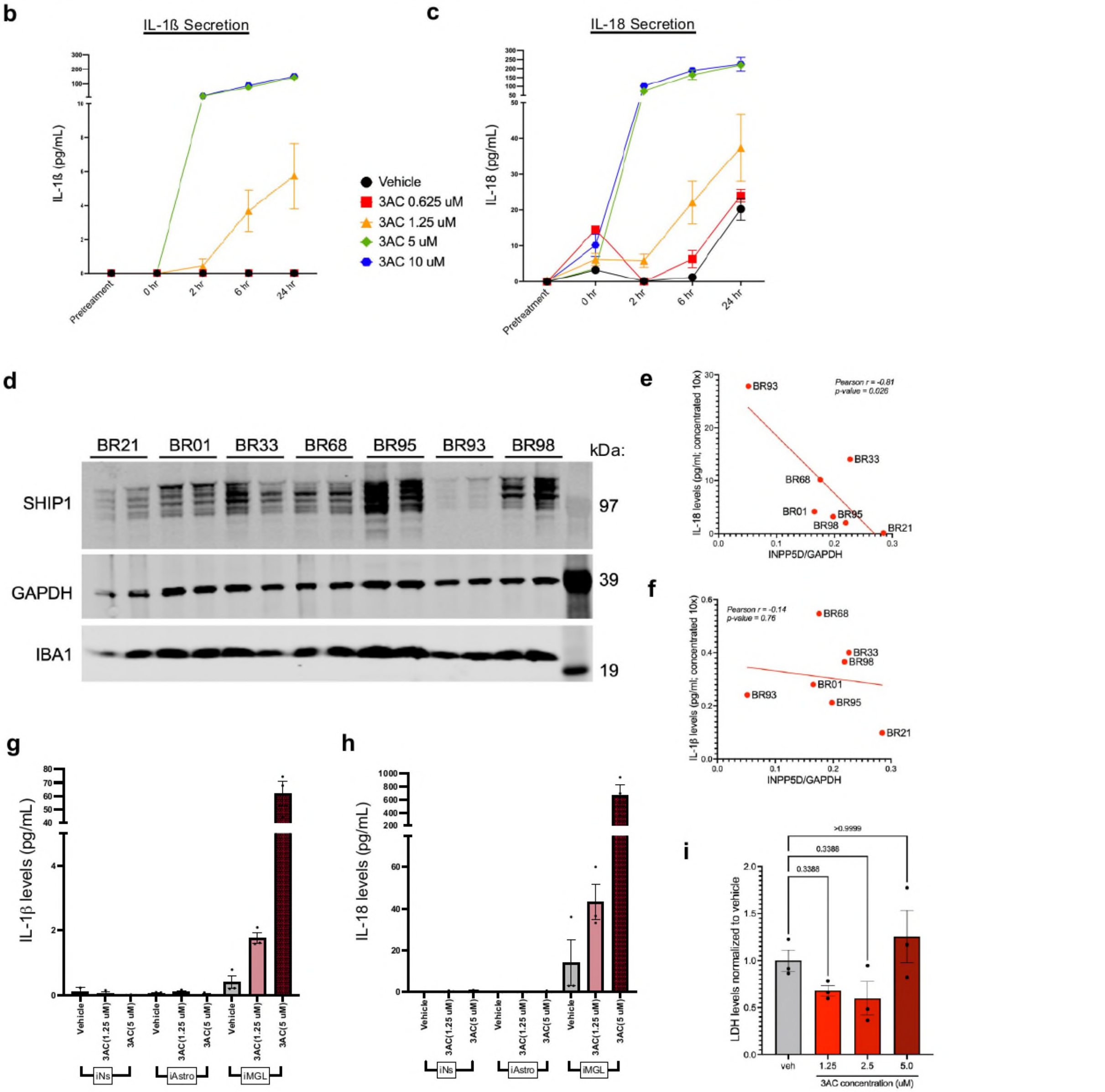

Supplementary Figure 7: 3AC treatment induces IL-1ß and IL-18 secretion in iMGs across multiple genetic backgrounds. a. A table of data regarding the 8 iPSC lines utilized, and the measured fold change of IL-1ß and IL-18 after 3AC (1.25µM 3AC, 6 hours) treatment. **b-c.** IL-1ß and IL-18 measured at various timepoints (0 hour, 2 hour, 6 hour, 24 hour) of BR33 iMGs treated with increasing concentration of 3AC (Vehicle, 0.625µM, 1.25µM, 5µM, 10µM 3AC). n = 3-4 wells per condition. **d.** Western blot of INPP5D protein levels across genotype. **e,f.** protein levels of INPP5D/GAPDH are graphed compared to secreted IL-18 (e) or IL-1 ß (f) by line. **g,h**. IL-1ß and IL-18 were measured using MSD ELISA following treatments. All samples were run on one ELISA plate to compare across cell type conditions. n = 3 wells per condition. Mean +/- SEM. nd = not detected, all samples were below detection range. **i.** LDH assay to examine cell death following vehicle or 3AC treatment. No significant differences were observed in cell death with 1.25, 2.5 or 5.0µM 3AC treatment of iMGs, although a trend of an increase in elevation in LDH was observed with 5uM 3AC. One-way ANOVA with Dunnett's multiple comparisons test, mean +/- SEM is shown, n = 3 per condition. For **i:** ns = p>0.05

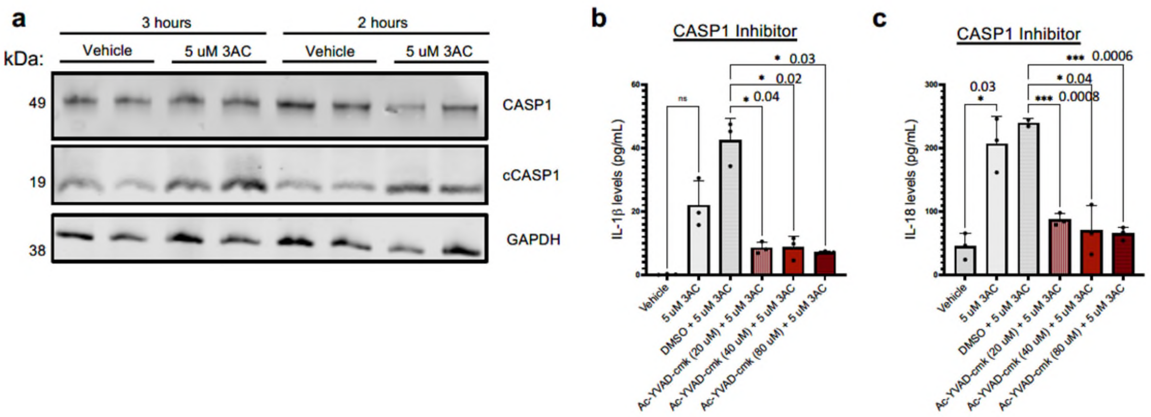

Supplementary Figure 8: Cleaved caspase 1 increases following INPP5D inhibition. a. iMGs were treated with either vehicle (ethanol) or 3AC (5µM) for 3 or 2 hours and harvested to collect protein. Caspase 1 and cleaved caspase 1 levels were assayed via western blotting. **b-c.** Treatment of iMGs with either vehicle (ethanol) or 5µM 3AC with either DMSO or Ac-YVADcmk (20µM, 40µM, 80µM) pre-treatment for 1 hour. Levels of secreted IL-1ß and IL-18 were measured using an MSD ELISA. n = 3 wells per condition. Mean +/- SEM. One-way ANOVA with Sidak's multiple comparison test. For **b-c:** ns = not significant, p>0.05, *p < 0.05, *** p < 0.001.

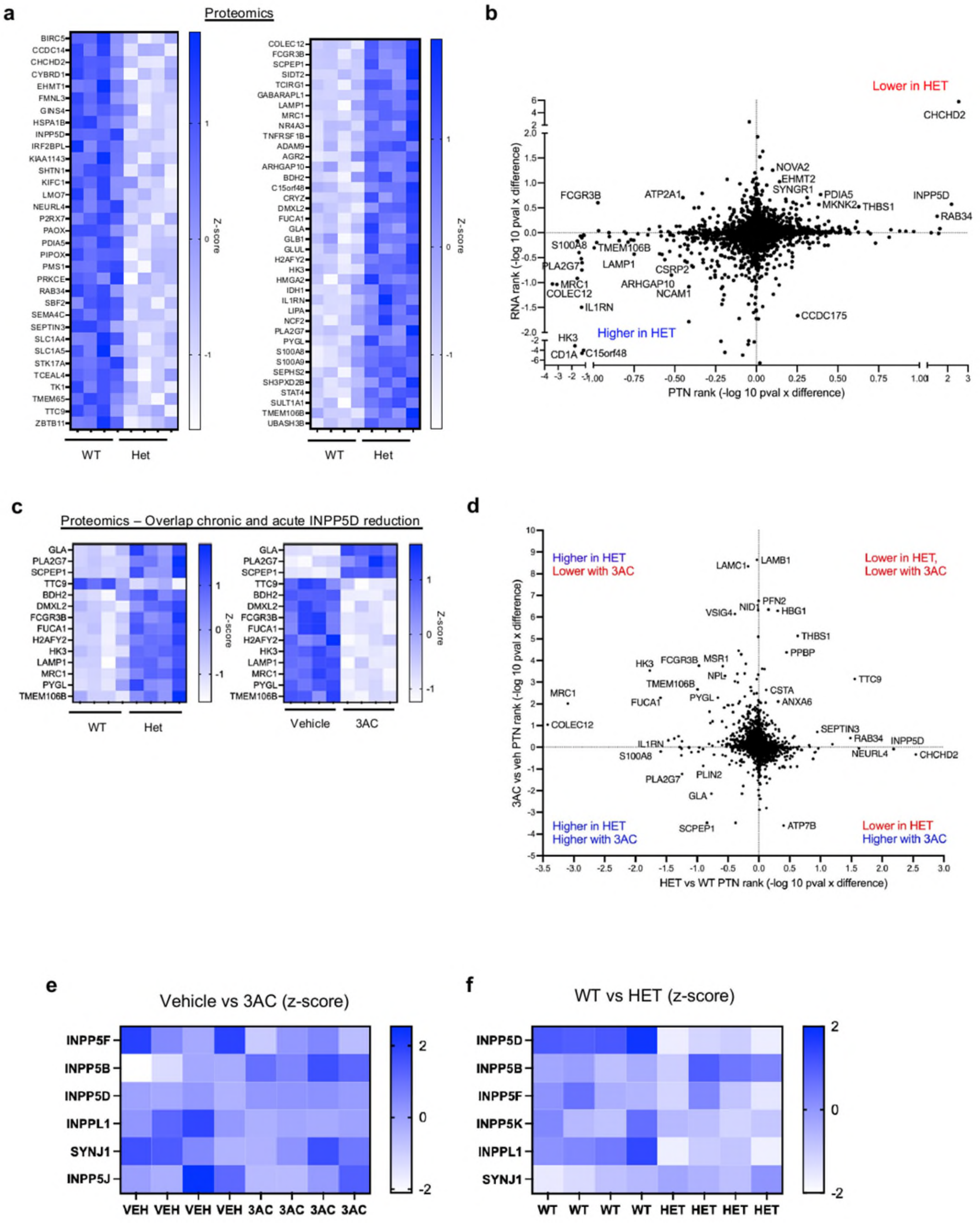

Supplementary Figure 9: Proteomic changes between INPP5D WT and HET iMGs. a. Heatmap of relative expression of all the differentially expressed proteins comparing INPP5D WT and HET iMGs (BH FDR<0.05). **b.** Comparison of DEG (RNAseq) and DEP (TMT-MS) analyses highlights genes concordantly and discordantly regulated with HET vs WT iMGs. Following HET vs WT comparisons (**Fig. 6a**), each gene was ranked by taking the -log10 of the p-value multiplied by the difference in expression between HET and WT samples. The rank in RNA versus protein-level analyses are graphed. **c.** Heatmap of relative expression of overlapping differentially expressed proteins between chronic decrease of INPP5D activity (WT and HET) and acute decrease of INPP5D activity (vehicle vs 3AC). Of note, many DEPs are in the opposite direction between acute and chronic INPP5D reduction, suggesting a potential feedback mechanism following 3AC treatment. **d.** Comparison of 3AC vs veh (DEP) and HET vs WT (DEP) analyses highlights genes concordantly and discordantly regulated with chronic and acute reduction in INPP5D levels or activity. Ranks for each genes for each comparison are shown. **e,f.** phosphoinositide family members are not differentially expressed with reduction in INPP5D levels or activity. Complete data sets found in Supplementary Data 17 and 19.

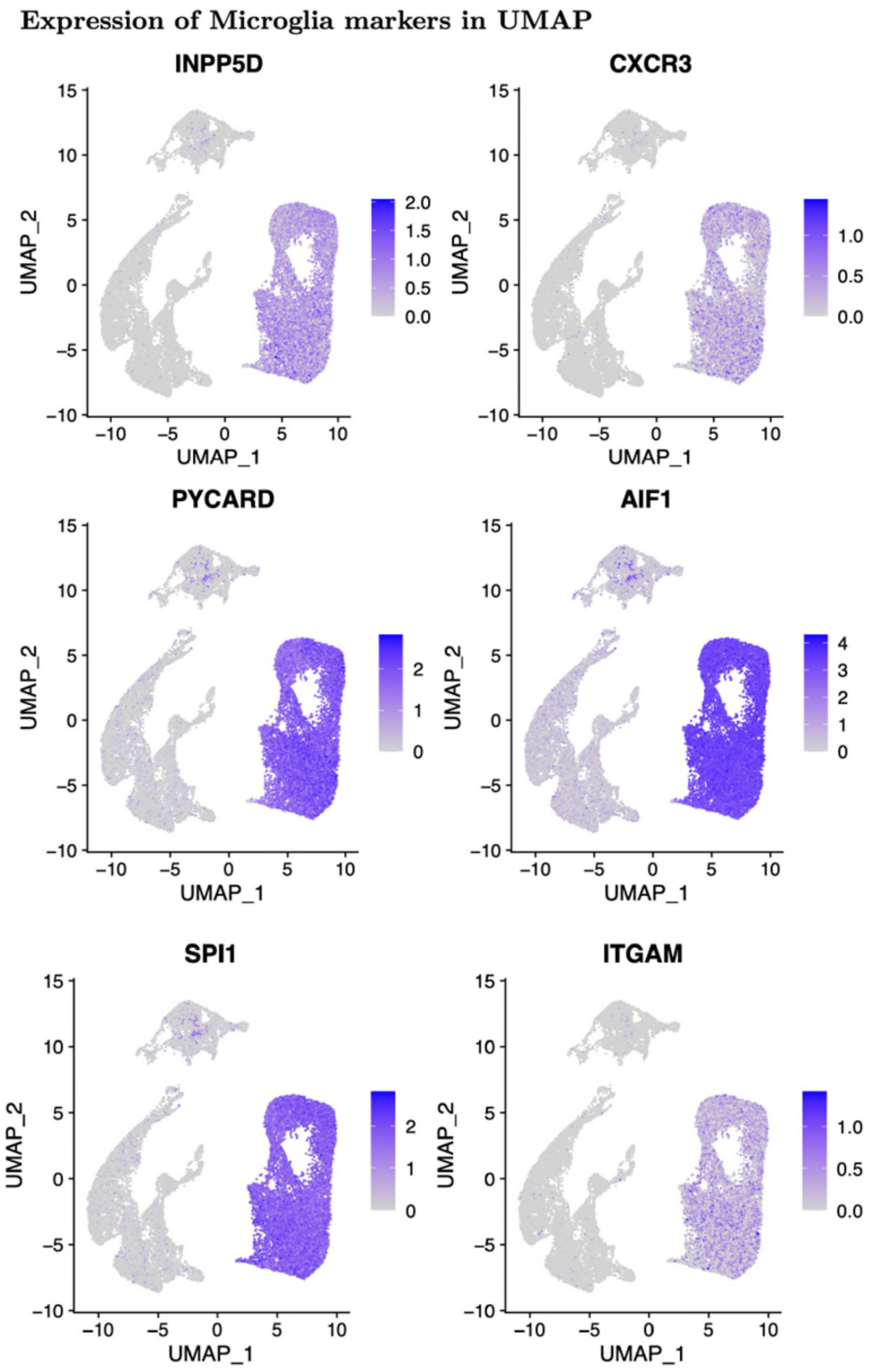

Supplementary Figure 10: Identification of microglial clusters in scRNAseq data. Shown are UMAP plots with expression of microglial cell fate markers, related to **Figure 8**.

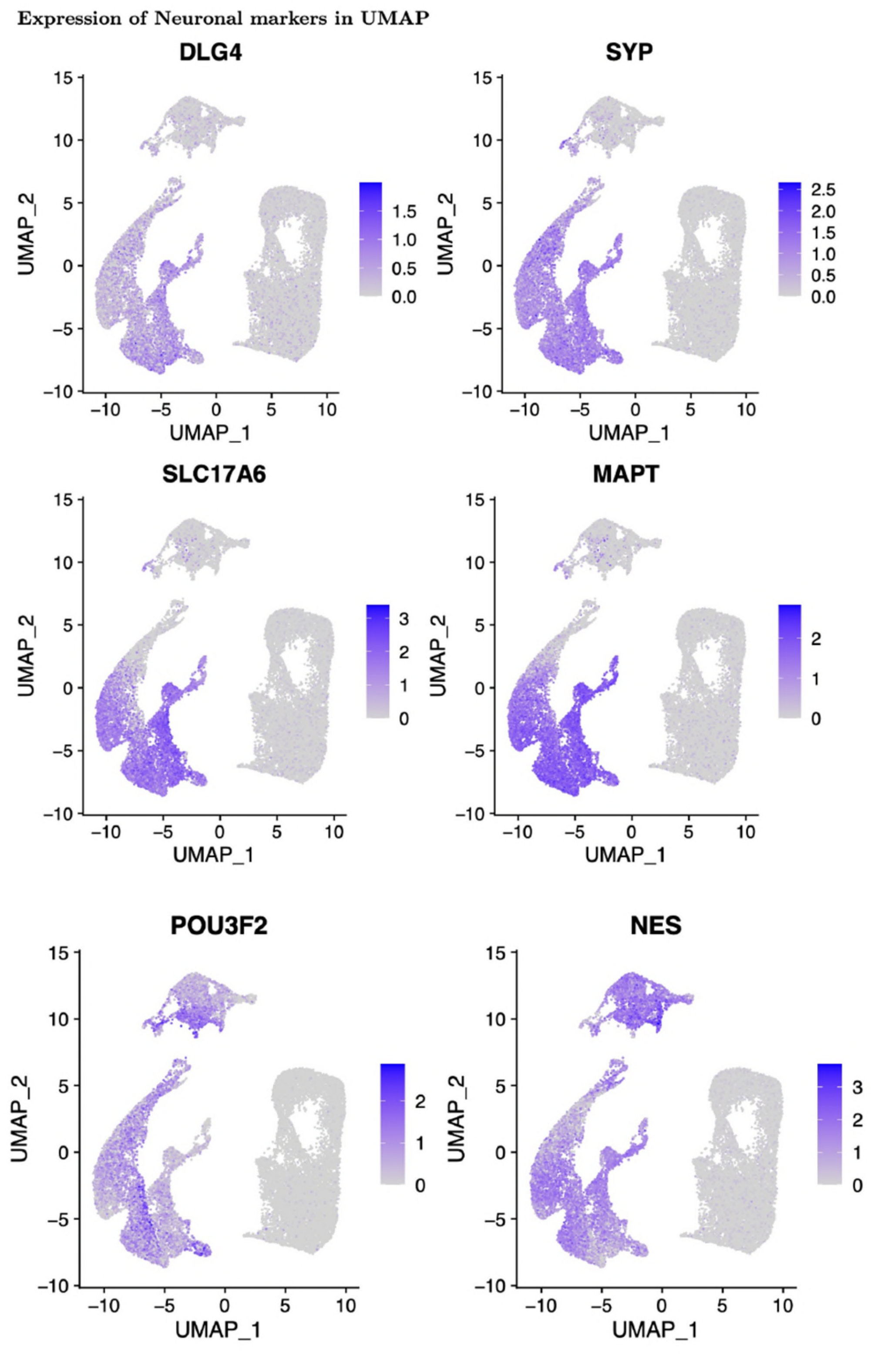

Supplementary Figure 11: Identification of neuron clusters in scRNAseq data. Shown are UMAP plots with expression of neuronal cell fate markers, related **to Figure 8**.

Top 10 NEG enriched pathways (higher in wt v. het exposed iNs)

NEG. Enriched Pathways
het v. wt. exposed d21 iNs

	padj	NES	size
GOCC_NEURON_PROJECTION	1.2e-15	-1.8	959
GOCC_AXON	4.7e-14	-2.0	498
GOBP_VESICLE_MEDIATED_TRANSPORT	1.9e-10	-1.6	1116
GOBP_SYNAPTIC_SIGNALING	2.2e-09	-1.8	497
GOCC_SYNAPTIC_MEMBRANE	2.4e-08	-1.9	271
GOCC_PRESYNAPSE	4.5e-08	-1.8	396
GOCC_GOLGI_APPARATUS	1.6e-07	-1.5	1168
GOCC_TRANSPORT_VESICLE	7.2e-07	-1.8	317
GOCC_SOMATODENDRITIC_COMPARTMENT	9.1e-07	-1.7	619
GOBP_CELLULAR_COMPONENT_MORPHOGENESIS	2.7e-06	-1.7	589

*Using full gene ontology gene list collection (cc, mf, bp) c5.go.v2022.1.Hs.symbols.gmt

Top 10 POS enriched pathways (higher in wt v. het exposed iNs)

POS. Enriched Pathways
het v. wt. exposed d21 iNs

	padj	NES	size
GOCC_RIBONUCLEOPROTEIN_COMPLEX	1.2e-19	1.9	656
GOMF_RNA_BINDING	1.2e-18	1.7	1437
GOBP_ORGANONITROGEN_COMPOUND_BIOSYNTHETIC_PROCESS	6.2e-16	1.7	1341
GOBP_PEPTIDE_BIOSYNTHETIC_PROCESS	6.2e-16	1.9	602
GOBP_AMIDE_BIOSYNTHETIC_PROCESS	2.7e-15	1.8	695
GOBP_PEPTIDE_METABOLIC_PROCESS	2.9e-15	1.8	707
GOBP_CELLULAR_MACROMOLECULE_BIOSYNTHETIC_PROCESS	1.2e-14	1.8	926
GOCC_RIBOSOME	1.2e-14	2.0	204
GOBP_CELLULAR_AMIDE_METABOLIC_PROCESS	1.6e-14	1.8	886
GOCC_RIBOSOMAL_SUBUNIT	3.6e-14	2.0	177

*Using full gene ontology gene list collection (cc, mf, bp) c5.go.v2022.1.Hs.symbols.gmt

Enrichment plot for top POS hit

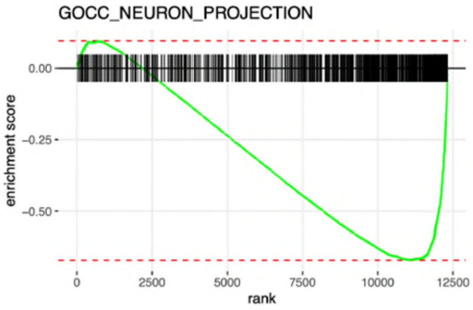

Enrichment plot for top hit

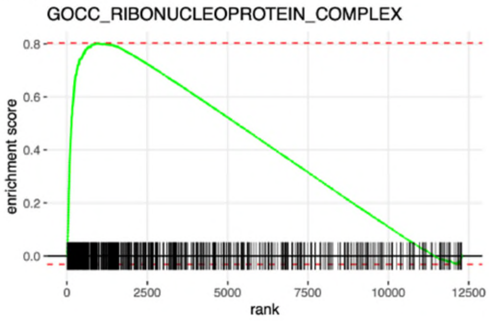

Supplementary Figure 12: GSEA results from scRNAseq data comparing iNs cocultured with WT iMGs or INPP5D HET iMGs.

Source data for Supplemental Figure 1

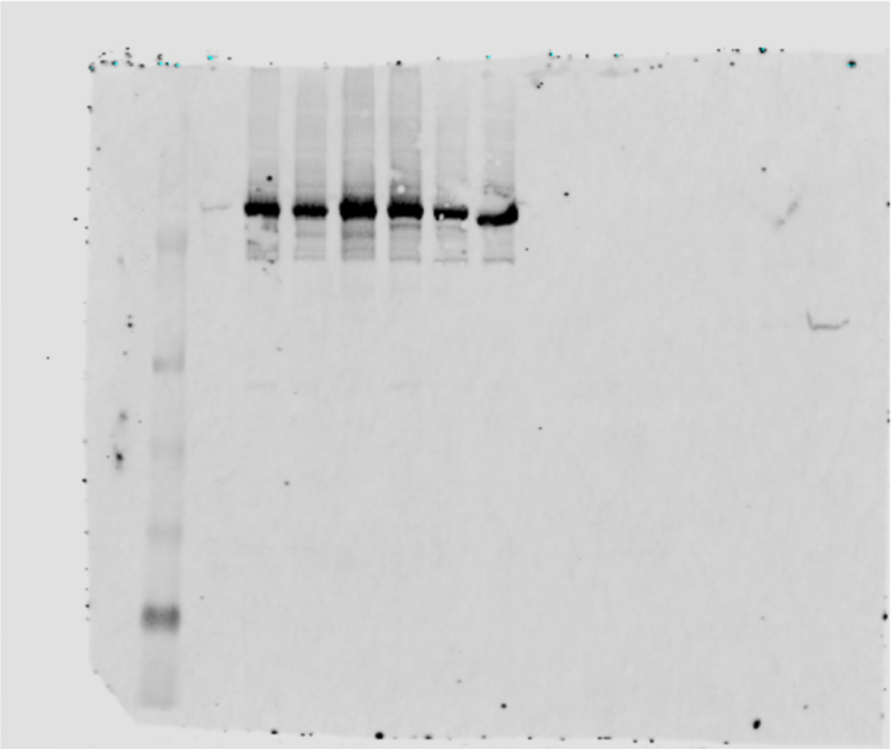

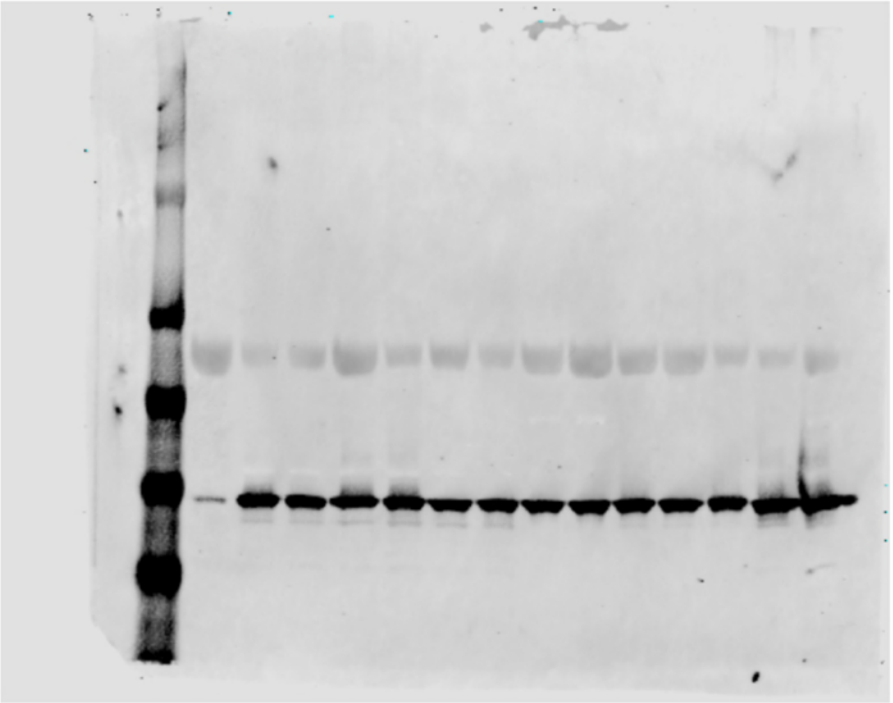

Source data for Supplemental Figure 7

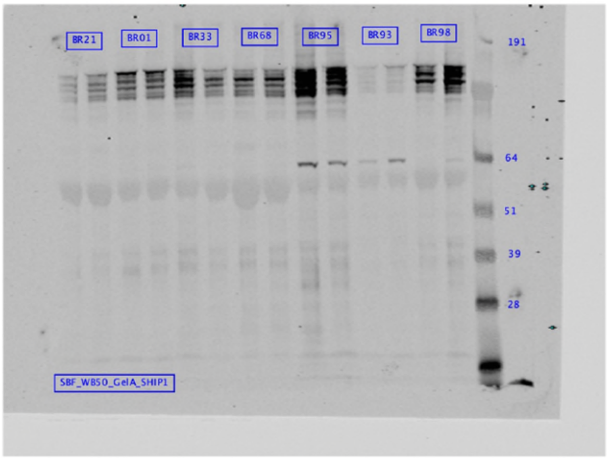

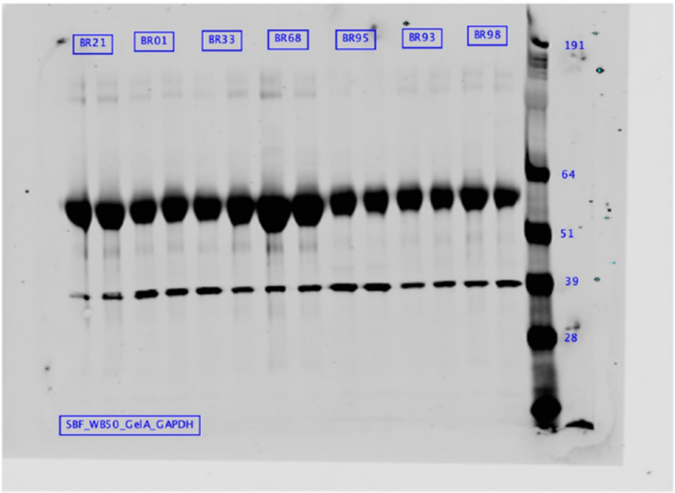

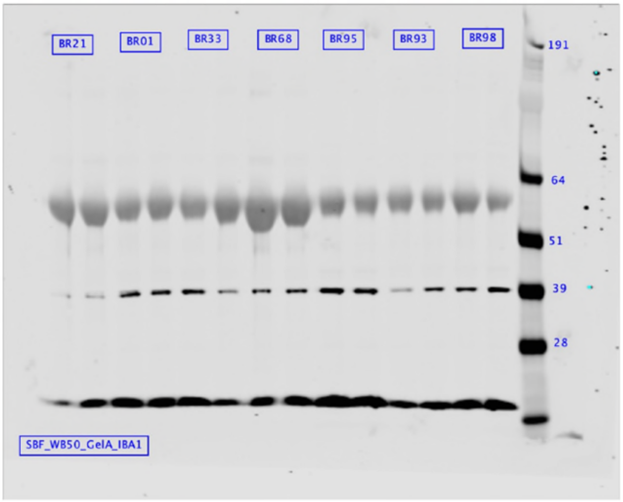

Source data for Supplemental Figure 8

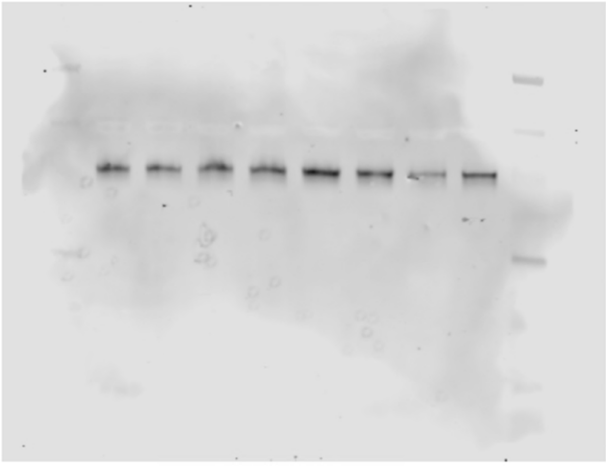

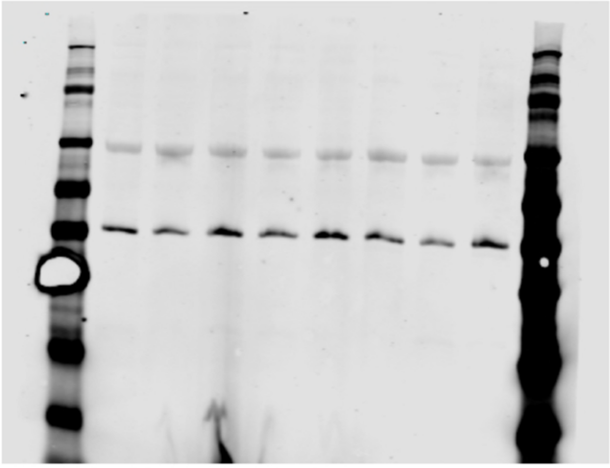

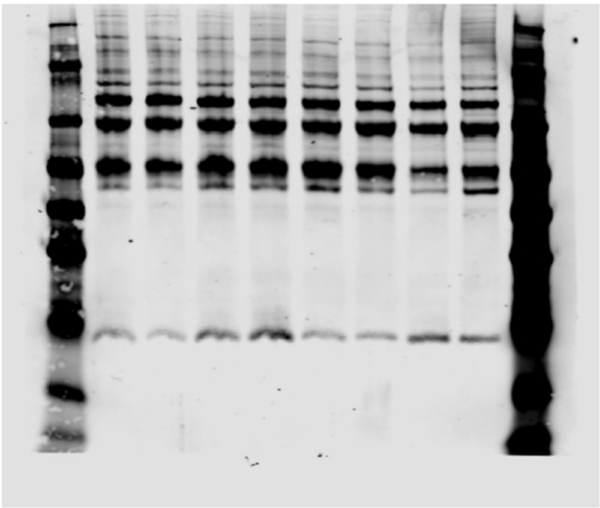

Supplement: Supplementary file 1 — Supplementary Information [file 41467_2023_42819_MOESM1_ESM.pdf]
