# Supplementary material for: INPP5D regulates inflammasome activation in human microglia
Source: Nat Commun. 2023 Nov 29;14:7552. doi: 10.1038/s41467-023-42819-w (PMC10684891; doi:10.1038/s41467-023-42819-w)
Supplement: Supplementary file 3 — Description of Additional Supplementary Files [file 41467_2023_42819_MOESM3_ESM.docx]

**Description of Additional Supplementary Files**

**File Name:** Supplementary Data 1

**Description:** Cluster enrichment for single nucleus RNAseq data from human brain. Shown is full list of cluster-enriched or depleted genes, derived from a Wilcoxon (2-sided) rank-sum test, as implemented in the Seurat FindAllMarkers function.

**File Name:** Supplementary Data 2

**Description:** INPP5D measurements for urea extracted brain tissue samples (DL-PFC, BA9) obtained from ROS and MAP cohorts measured in this study.

**File Name:** Supplementary Data 3

**Description:** Microglial-level analysis of immunocytochemistry data obtained from brain tissue regarding shape, plaque-association, INPP5D levels; see Supplementary Data 13 for metadata.

**File Name:** Supplementary Data 4

**Description:**Expression matrix of RNAseq data from iMGLs; see also Supplementary Data 14 for metadata.

**File Name:** Supplementary Data 5

**Description:**Protein abundance table for iMGLs treated with vehicle or 3AC or else comparing WT versus INPP5D het. See also Figures 3 and 6.

**File Name:** Supplementary Data 6

**Description**: RNAseq expression matrix for WT iMGs and INPP5D biallelic loss-of-function iMGs.

**File Name:** Supplementary Data 7

**Description:** RNAseq expression matrix for iMGs transduced with empty lentivirus or INPP5D overexpression (OE) lentivirus.

**File Name:** Supplementary Data 8

**Description:** Summary of all the cell lines used throughout this study. Lines with a BRID were generated from a previous study. ROS = Religious Order Study, MAP = Memory and Aging Project, LP-NCI = Low pathology and not cognitively impaired, AD = Alzheimer's disease, NA = not available.

**File Name:** Supplementary Data 9

**Description:** Summary table of all the TBS Brain extracts. LPNCI = Low Pathology, Not Cognitively Impaired, HPNCI = High Pathology and Not-Cognitively Impaired, AD = Alzheimer's disease, NA = No data available.

**File Name:** Supplementary Data 10

**Description:** Summary table of urea brain extracts. AD = Alzheimer's Disease, MCI = Mild Cognitive Impairment, LPNCI = Low Pathology, Not Cognitively Impaired, HPNCI = High Pathology and Not-Cognitively Impaired. Dcfdx=clinical diagnosis, pm=clincial AD diagnosis, pathoAD =pathological AD diagnosis.

**File Name:** Supplementary Data 11

**Description:**Relevant data for TBS-soluble brain tissue samples measured in this study. ND=not determined due to limited tissue availability; NA=not applicable

**File Name:** Supplementary Data 12

**Description:** TMT-MS data from from Johnson et al, 2022 <https://doi.org/10.1038/s41593-021-00999-y> fr INPP5D. ND=not determined due to limited tissue availability; NA=not applicable.

**File Name:** Supplementary Data 13

**Description:** Metadata for Brain samples obtained from the NYBB for immunocytochemistry.

**File Name:** Supplementary Data 14

**Description:** Metadata for RNAseq of iMGLs in Supplementary Data 4. See also Figure 3.

**File Name:** Supplementary Data 15

**Description:** Primers and sgRNA sequences for this study.

**File Name:** Supplementary Data 16

**Description:** Brain-level quantification of ASC, plaque, and INPP5D ICC data from the human brain.

**File Name:** Supplementary Data 17

**Description:** Sleuth was employed to identify DEGs: differential expression was calculated using a 2-sided Wald test on normalized expression values. All q-values were calculated using the Benjamini Hochberg procedure. DEG results comparing iMGs that are WT versus bi-allelic loss-of-function of INPP5D.

**File Name:** Supplementary Data 18

**Description:** Sleuth was employed to identify DEGs: differential expression was calculated using a 2-sided Wald test on normalized expression values. All q-values were calculated using the Benjamini Hochberg procedure. DEG results comparing iMGs that are control versus overexpression INPP5D

**File Name:** Supplementary Data 19

**Description:** Sleuth was employed to identify DEGs: differential expression was calculated using a 2-sided Wald test on normalized expression values. All q-values were calculated using the Benjamini Hochberg procedure. DEG results comparing AD to NCI microglia, using pseudobulk data generated from ROSMAP brain tissue.

**File Name:** Supplementary Data 20

**Description:** Results of GSEA analyzing three RNAseq datasets (INPP5D overexpression, bi-allelic loss-of-function, and AD vs NCI microglia pseudobulk from brain tissue). Rank files were generated based upon DEG outputs in Supplemental Tables 21-23; ranks were calculated by taking signed the -log10 of the p-values. Shown are all enriched pathways arising with p<0.05 in at least 2 of the 3 comparisons. Relevant to Figure 7k.

**File Name:** Supplementary Data 21

**Description:** DEGs by cluster comparing WT iMGs to INPP5D HET iMGs or else iNs co-cultured with WT iMGs to INPP5D HET iMGs. DEGs calculated by Wilcoxon Rank Sum test (2-sided) with a multiple comparisons adjusted (FDR) p-value cutoff of 0.05, and LogFC threshold of 0.25.

**File Name:** Supplementary Data 22

**Description:** DEGs of scRNAseq data of WT iMGs versus INPP5D HET iMGs (Figure 8). DEGs calculated by Wilcoxon Rank Sum test (2-sided) with a multiple comparisons adjusted (FDR) p-value cutoff of 0.05, and LogFC threshold of 0.25.

**File Name:** Supplementary Data 23

**Description:** DEGs of scRNAseq data of iNs co-cultured with WT iMGs versus INPP5D HET iMGs (Figure 8). DEGs calculated by Wilcoxon Rank Sum test (2-sided) with a multiple comparisons adjusted (FDR) p-value cutoff of 0.05, and LogFC threshold of 0.25.

**File Name:** Supplementary Data 24

**Description:** Composition of clusters in scRNAseq data shown in Figure 8.
