# Supplementary material for: INPP5D regulates inflammasome activation in human microglia
Source: Nat Commun. 2023 Nov 29;14:7552. doi: 10.1038/s41467-023-42819-w (PMC10684891; doi:10.1038/s41467-023-42819-w)
Supplement: Supplementary file 5 — Reporting Summary [file 41467_2023_42819_MOESM5_ESM.pdf]

nature portfolio

Corresponding author(s): Tracy Young-Perase

Last updated by author(s): September 27. 2023

Reporting Summary

Nature Portfolio wishes to improve the reproducibility of the work that we publish. This form provides structure for consistency and transparency in reporting. For further information on Nature Portfolio policies, see our Editorial Policies and the Editorial Policy Checklist.

Statistics

For all statistical analyses, confirm that the following items are present in the figure legend, table legend, main text, or Methods section.

n/a	Confirmed	
☐	☒	The exact sample size (*n*) for each experimental group/condition, given as a discrete number and unit of measurement
☐	☒	A statement on whether measurements were taken from distinct samples or whether the same sample was measured repeatedly
☐	☒	The statistical test(s) used AND whether they are one- or two-sided<br>*Only common tests should be described solely by name; describe more complex techniques in the Methods section.*
☐	☒	A description of all covariates tested
☐	☒	A description of any assumptions or corrections, such as tests of normality and adjustment for multiple comparisons
☐	☒	A full description of the statistical parameters including central tendency (e.g. means) or other basic estimates (e.g. regression coefficient) AND variation (e.g. standard deviation) or associated estimates of uncertainty (e.g. confidence intervals)
☐	☒	For null hypothesis testing, the test statistic (e.g. *F*, *t*, *r*) with confidence intervals, effect sizes, degrees of freedom and *P* value noted<br>*Give P values as exact values whenever suitable.*
☒	☐	For Bayesian analysis, information on the choice of priors and Markov chain Monte Carlo settings
☒	☐	For hierarchical and complex designs, identification of the appropriate level for tests and full reporting of outcomes
☒	☐	Estimates of effect sizes (e.g. Cohen's *d*, Pearson's *r*), indicating how they were calculated

Our web collection on statistics for biologists contains articles on many of the points above.

Software and code

Policy information about availability of computer code

Data collection	Seurat v4.0.3 package for R, 10x Genomics CellRanger pipeline "filtered_feature_bc_matrix", Harmony 0.1.1, Kallisto pseudoalignment quantification program (v0.43.1), "Sleuth" package (v0.30.0) in R Studio (v3.6.1 of R; v1.2.5019 of R Studio), limma v3.54.2,
Data analysis	Prism version 9, R Studio, v3.6.1 of R; v1.2.5019 of R Studio, Kallisto pseudoalignment quantification program, v0.43.1, Sleuth, v0.30.0, ComBat algorithm in the SVA (v3.34.0) package in R, MaxQuant v1.6.3.4, Thermo's Proteome Discoverer suite (version 2.4.1.15) with Sequest HT, SVA 3.46.0, "DEP" package in R (v1.20.0), Seurat package (v4.0.4) in R (v4.0.3) using RStudio (v1.4.1103)

For manuscripts utilizing custom algorithms or software that are central to the research but not yet described in published literature, software must be made available to editors and reviewers. We strongly encourage code deposition in a community repository (e.g. GitHub). See the Nature Portfolio guidelines for submitting code & software for further information.

Data

Policy information about availability of data

All manuscripts must include a data availability statement. This statement should provide the following information, where applicable:

- Accession codes, unique identifiers, or web links for publicly available datasets
- A description of any restrictions on data availability
- For clinical datasets or third party data, please ensure that the statement adheres to our policy

A thorough DATA AVAILABILITY section is included, and copied here:
The RNAseq data generated in this study have been deposited in the NCBI-GEO database under accession code (GSE244209). The proteomic data generated in this study are provided in the Supplementary Information file. All source data not presented in Supplemental Tables are provided within the Source Data file. An interactive data viewer for RNAseq and proteomic data presented in this study from iPSC-derived microglia also can be found here: https://

youngpearselab.shinyapps.io/inpp5d_imgls/
Additional phenotypic data from ROS and MAP cohorts can be requested at www.radc.rush.edu. Previously published data from brain tissue data (citations found within) can be found on the AMP-AD Knowledge Portal:
Genotype data: doi:10.1038/mp.2017.20.
RNAseq: doi:10.1038/s41593-018-0154-9.
TMT-MS: doi:10.1101/806752

Field-specific reporting

Please select the one below that is the best fit for your research. If you are not sure, read the appropriate sections before making your selection.

☒ Life sciences ☐ Behavioural & social sciences ☐ Ecological, evolutionary & environmental sciences

For a reference copy of the document with all sections, see nature.com/documents/nr-reporting-summary-flat.pdf

Life sciences study design

All studies must disclose on these points even when the disclosure is negative.

Sample size	For cell culture experiments, sample sizes were chosen based upon our previous studies using iPSC derivatives, which always include at least 3 separate differentiations and 3 biological replicates per condition per experiment (doi: 10.1038/s41380-022-01454-5.; doi: 10.1016/j.neuron.2021.08.003.; doi: 10.1016/j.stemcr.2022.08.001;doi: 10.1016/j.celrep.2023.112994). For brain analyses, numbers were determined based upon availability of existing published data or else on availability of brain tissue and our previous experience in associating expression levels with genotypes (doi: 10.1016/j.neuron.2021.08.003; doi:10.1101/806752; doi:10.1038/s41593-018-0154-9).
Data exclusions	Data was not excluded from analyses unless otherwise stated in the methods or figure legends.
Replication	In vitro experiments were repeated across at least 3 separate differentiations and 3 biological replicates per condition per experiment, and across multiple cell lines. Both chemical and genetic perturbation of INPP5D were used to identify inflammasome activation as relevant to INPP5D biology. All attempts at replication are included herein.
Randomization	Within in vitro experiments, wells were randomized to treatment conditions.
Blinding	Blinding was relevant to our study for the instance of examining ASC spec formation in iMGLs with positive controls, negative controls, and 3AC treatment. For brain WBs, experimenter was blind to diagnosis for the running and quantification of the Western blots. For brain ICC, experimenter was blind to diagnosis through image collection.

Reporting for specific materials, systems and methods

We require information from authors about some types of materials, experimental systems and methods used in many studies. Here, indicate whether each material, system or method listed is relevant to your study. If you are not sure if a list item applies to your research, read the appropriate section before selecting a response.

Materials & experimental systems

n/a	Involved in the study	
☐	☒	Antibodies
☐	☒	Eukaryotic cell lines
☒	☐	Palaeontology and archaeology
☒	☐	Animals and other organisms
☐	☒	Human research participants
☒	☐	Clinical data
☒	☐	Dual use research of concern

Methods

n/a	Involved in the study	
☒	☐	ChIP-seq
☒	☐	Flow cytometry
☒	☐	MRI-based neuroimaging

Antibodies

Antibodies used

Anti-SHIP1/INPP5D (C40G9) Rabbit antibody (Cell Signaling Technologies, 2727S) for immunostaining (1:100) and western blotting (1:500)
Anti-SHIP1/INPP5D (ab45142), abcam for immunostaining (1:100) and western blotting (1:1000)
Anti-P2RY12 Rabbit antibody (from Burtovsky lab), for immunostaining (1:500)
Anti-GAPDH Mouse antibody (Proteintech, MAB374), for western blotting (1:10,000)
Anti-Iba1 antibody (abcam, ab5076) for immunostaining (1:500) and western blotting (1:500)
Anti-PAFAH Polyclonal Rabbit antibody (Proteintech, 15526-1-AP), for western blotting (1:500)
Anti-Mannose Receptor antibody (abcam, ab64693), for western blotting (1:500)
Anti-CL-P1/COLEC12 mouse antibody (R&D Systems, R&D AF3130), for western blotting (1:500)

Anti-Caspase-1 (D7F10) Rabbit antibody (Cell Signaling Technologies, 3866S), for western blotting (1:1000)
Anti-ASC/TMS1 (E1E3I) Rabbit antibody (Cell Signaling Technologies, 13833S), for western blotting (1:1000)
Anti-NLRP3 (D4D8T) Rabbit antibody (Cell Signaling Technologies, 15101S), for western blotting (1:1000)
Anti-Aβ42 (Biolegend, 805501), for immunostaining (1:300)
Anti-GSDMD (abcam, ab210070), for western blotting (1:1000)
Anti-LC3 (MBL International Corporation, M186-3),for western blotting (1:1000)

Validation

Specificity of INPP5D/SHIP1 antibodies were validated herein by using INPP5D knock out cell lines for the same applications. CiteAb was used to identify well validated antibodies when possible: GAPDH (3,70 citations), IBA1 (1,4440 citations); Mannose Receptor (817 citations); Caspase-1 (184 citations); ASC1 (55 citations); NLRP3 (469 citations); Aβ42 (29 citations), GSDMD (75 citations); LC3 (31 citations)
References validating less commonly employed antibodies utilized are presented here: P2RY12 (doi: 10.1038/nn.3599); PLA2G7 (doi: 10.3390/ijms24010882).

Eukaryotic cell lines

Policy information about cell lines

Cell line source(s)

iPSC lines from the ROS and MAP cohorts generated and characterized in the Young-Pearse lab and NYCSF (Lagomarsino et al, Neuron, 2021), details regarding each line used are listed in Supplemental Table 1 (sex, age at death. diagnosis and genotype), and lines are listed here:
BR01 - Female
BR09 - Male
BR21 - Female
BR22 - Female
BR24 - Female
BR33 - Male
BR68 - Male
BR65 - Female
BR93 - Male
BR95 - Female
BR98 - Male
BR99 - Female
BR103 - Female
BR33.1 (WT) - Male
BR33.6 (INPP5D 7bp del) - Male
BR33.7 (INPP5D 10bp del) - Male
BR33.47 (WT) - Male
BR33.50 (INPP5D 5bp del) - Male
BR33.78 (WT) - Male
BR33.80 (INPP5D 11bp ins) - Male
BR24.9 (INPP5D 2 bp ins; 15 bp del) - Female
BR24.12 (INPP5D 1bp ins; 5bp del) - Female

Authentication

Cell lines were repeatedly STR profiled throughout the study to confirm identity against profiles from PBMCs of the individuals from whom they were generated.

Mycoplasma contamination

All cell lines were mycoplasma negative and undergo monthly testing for mycoplasma contamination.

Commonly misidentified lines
(See ICLAC register)

No commonly misidentified lines were used in this study.

Human research participants

Policy information about studies involving human research participants

Population characteristics	Population characteristics included in the supplemental tables: sex, age at death, clinical and pathological diagnosis of Alzheimer's dementia and disease, APOE genotype status, INPP5D genotype status. Citations are included that describe how diagnoses were obtained. The following sex distributions were used for the following experiments: single-nucleus sequencing of whole brain (6 male, 6 female), immunostaining of human brain samples (7 male, 9 female), TBS-soluble human brain extracts (33 male, 59 female), urea-soluble human brain extracts (78 male, 116 female)
Recruitment	The ROS and MAP cohorts were previously reported and data collected and published regarding recruitment.
Ethics oversight	All work was performed following IRB review and approval through Partners/BWH IRB (2016P000867). Human brain material was obtained from 1) the neuropathology core facility at Massachusetts General Hospital, 2) Rush University Medical Center, 3) Albany Medical Center, and 4) New York Brain Bank. ROS and MAP studies were approved by an Institutional Review Board of Rush University Medical Center. All participants signed an informed consent, an Anatomical Gift Act, and a repository consent to allow their data and biospecimens to be repurposed, and for data acquired from these samples to be published. The ROSMAP studies were approved by an institutional review board of Rush University Medical Center. Each participant signed an informed consent, an Anatomical Gift Act for organ donation, and a separate repository consent allowing for sharing and repurposing of data and biospecimens. IPSC lines were utilized following IRB review and approval through MGB/BWH IRB (#2015P001676).

Note that full information on the approval of the study protocol must also be provided in the manuscript.
